# Phosphorylation of Guar Gum/Magnetite/Chitosan Nanocomposites for Uranium (VI) Sorption and Antibacterial Applications

**DOI:** 10.3390/molecules26071920

**Published:** 2021-03-29

**Authors:** Mohammed F. Hamza, Amr Fouda, Khalid Z. Elwakeel, Yuezhou Wei, Eric Guibal, Nora A. Hamad

**Affiliations:** 1 Guangxi Key Laboratory of Processing for Non-Ferrous Metals and Featured Materials, School of Resources, Environment and Materials, Guangxi University, Nanning 530004, China; m_fouda21@hotmail.com; 2 Nuclear Materials Authority, POB 530, El-Maadi, Cairo 11884, Egypt; 3 Botany and Microbiology Department, Faculty of Science, Al-Azhar University, Nasr City, Cairo 11884, Egypt; amr_fh83@azhar.edu.eg; 4 Department of Chemistry, College of Science, University of Jeddah, Jeddah 80327, Saudi Arabia; kelwkeel@uj.edu.sa; 5 Environmental Science Department, Faculty of Science, Port-Said University, Port-Said 42522, Egypt; 6 School of Nuclear Science and Engineering, Shanghai Jiao Tong University, Shanghai 200240, China; 7 Polymers Composites and Hybrids (PCH), IMT Mines Ales, F-30319 Alès, France; 8 Faculty of Science, Menoufia University, Shebine El-Koam 00123, Egypt; nhamad059@gmail.com

**Keywords:** guar-gum phosphorylation, chitosan-based composite, magnetite nanoparticles, uranyl sorption and desorption, sorption isotherms and uptake kinetics, selectivity, antibacterial activity

## Abstract

The development of new materials is needed to address the environmental challenges of wastewater treatment. The phosphorylation of guar gum combined with its association to chitosan allows preparing an efficient sorbent for the removal of U(VI) from slightly acidic solutions. The incorporation of magnetite nanoparticles enhances solid/liquid. Functional groups are characterized by FTIR spectroscopy while textural properties are qualified by N_2_ adsorption. The optimum pH is close to 4 (deprotonation of amine and phosphonate groups). Uptake kinetics are fast (60 min of contact), fitted by a pseudo-first order rate equation. Maximum sorption capacities are close to 1.28 and 1.16 mmol U g^−1^ (non-magnetic and magnetic, respectively), while the sorption isotherms are fitted by Langmuir equation. Uranyl desorption (using 0.2 M HCl solutions) is achieved within 20–30 min; the sorbents can be recycled for at least five cycles (5–6% loss in sorption performance, complete desorption). In multi-component solutions, the sorbents show marked preference for U(VI) and Nd(III) over alkali-earth metals and Si(IV). The zone of exclusion method shows that magnetic sorbent has antibacterial effects against both Gram+ and Gram- bacteria, contrary to non-magnetic material (only Gram+ bacteria). The magnetic composite is highly promising as antimicrobial support and for recovery of valuable metals.

## 1. Introduction

The recovery of valuable metals and the removal of hazardous metal ions is an important challenge for maintaining competitive supply of metals under environmentally acceptable conditions. Conventional processes consist of metal precipitation [1], solvent extraction [2,3,4], and membrane-based techniques [5,6], which may face some limitations depending on the concentration levels, the complexity of the solutions, the target levels of discharge, or the flow rates involved in industrial processes. Resins and sorbents are more specifically preferred for the treatment of low-metal concentrations effluents. 

Despite the large range of commercially available resins, there is still a need for developing alternative sorbents for both the removal of hazardous metal ions and the recovery of valuable metals. Uranium is an emblematic example of hazardous metals that require removal from contaminated effluents and underground water; in addition, the demand on uranium for feeding the nuclear power industry is another motivation for designing such new materials. Biosorption has attracted a great deal of attention over the last decades for the removal of metal ions from water [7,8,9]. Mimicking the conventional industrial resins that were designed with specific functional groups, biosorption aims to use bio-based materials (biomass, biopolymers) naturally bearing analogue functional groups (as those bear by industrial resins), chemically modified by grafting specific reactive groups, or composites based on biopolymers [10,11].

In the case of uranium, a wide variety of reactive groups has been identified for their affinity for the metal not only based on high sorption capacities but also on selectivity (for recovery from highly saline solutions):

(a)  Amidoxime groups immobilized on synthetic resins [12,13,14], metal-organic frameworks [15], or bio-based composites [16,17]
(b)  Sulfonic acid-bearing materials [18,19,20],
(c)  Quaternary ammonium-based sorbents [21,22,23],
(d)  Iminodiacetic acid-bearing groups [24].

However, during the last decades, a great effort has been focused on the development of phosphorus-based resins and sorbents for enhancing the recovery of uranium from aqueous solutions, industrial effluents, and seawater [25,26,27,28,29,30]. Several studies have pointed out the beneficial effect of the bi-functionality of phosphorus-based resins for U(VI) uptake [31,32,33,34,35,36,37,38]. More specifically, aminophosphonate-bearing resins showed high affinity for uranium [39,40,41,42]. This rationale has driven the research developed in the present work. The concept is based on the use of a biopolymer-based support for designing a composite amino-bearing sorbent and its phosphorylation. Herein, the composite consists of the phosphorylation of guar gum (an abundant galactomannan polysaccharide, β-1,4-linked mannose residues to which galactose residues are 1,6-linked to form short sidechains), prior to its linkage with chitosan (deacetylated form of chitin, the main constituent of the exoskeleton of crustaceans, insect cuticule, and fungal wall). The phosphorylation is based on the reaction of hydroxyl groups on guar gum with phosphoric acid in the presence of urea (following a method described for the phosphorylation of alginate, [43]). The grafting of chitosan to phosphorylated guar gum offers the presence of both amino and phosphonate groups. Chitosan has shown a high affinity for the sorption of metal ions through chelation of metal cations in near-neutral solutions and electrostatic attraction/ion exchange mechanisms in acidic solutions for the binding of metal anions [44,45,46,47,48]. Guar gum has also retained a great attention in the last decades for synthesizing metal-binding composite hydrogels [49,50,51,52,53,54].

Frequently the sorption performance of these biopolymer-based hydrogels is limited by mass transfer properties, especially after uncontrolled drying (freeze-drying or drying under supercritical CO_2_ conditions allow overcoming these limitations). It is thus necessary decreasing the size of the particles to enhance uptake kinetics, at the expense of difficulties in practically processing the sorption operations (more specifically proceeding the solid/liquid separation after sorbent saturation). This problem can be overcome by incorporation of magnetite nanoparticles for improving the ready separation of spent sorbent from treated solution [16,48,55,56,57,58,59] using external magnetic field. 

In another field, the uncontrolled use of antibiotics led to biochemicals and genetic changes of bacterial strains (antibiotic-resistant) [60]; therefore, there is a clear necessity to develop new antibiotics and/or alternative strategies to prevent and cure microbial contaminations. Nanoobjects have recently shown promising bactericidal activity [61,62].

The study compares U(VI) sorption properties for functionalized composites (phosphorylated gum guar deposited on chitosan, both as non-magnetic and magnetic particles, PGG@C and PGG@MC, respectively) with the sorption performance of the precursors (i.e., chitosan (Chit); guar gum (GG), with or without incorporation of magnetite nanoparticles). After characterization of the sorbents by FTIR spectroscopy, textural (BET) and morphological (TEM, SEM, and SEM-EDX) characterizations, thermogravimetric analysis (TGA), their sorption properties for U(VI) binding are investigated through the study of pH effect, uptake kinetics, sorption isotherms, selectivity (sorption from multi-component solutions), metal desorption and sorbent recycling. In order to evaluate the feasibility of using these materials for industrial application, the sorbents are tested on real metal-bearing solutions: the effect of the complexity of the solutions can be measured on the sorption performance. In parallel to this application, complementary preliminary tests are performed for evaluate the antimicrobial effect of these materials (PGG@C and PGG@MC) against Gram+ bacteria (i.e., *Staphylococcus aureus* and *Bacillus subtilis*). Indeed, phosphonate derivatives have been reported to have direct (or assisting) antimicrobial activity [63]; in addition, to the positive effect of magnetic/chitosan nanoparticles [61,64].

## 2. Materials and Methods

### 2.1. Materials

All chemicals used in this study were analytical grade. Epichlorohydrin (EPI), ferrous sulfate (FeSO_4_·7H_2_O), chitosan (Chit, degree of acetylation, DA: <25%), ammonium ferric (III) sulfate dodecahydrate ((NH_4_)Fe(SO_4_)_2_·12H_2_O), sodium hydroxide, phosphoric acid, Muller-Hinton agar (microbiological growth medium), and dimethylsulfoxide (DMSO) were purchased from Sigma Aldrich (Merck KGAa, Darmstadt, Germany). Uranyl nitrate hexahydrate (UO_2_(NO_3_)_2_·6H_2_O) was obtained from Spi Supplies (West Chester, PA, USA). Dimethylformamide (DMF) and acetone were purchased from Chron Chemicals (Qionglai, China). Guar gum (GG) was obtained from Alpha Chemika (Mumbai, Maharashtra, India). Urea was supplied by Chem Lab (Zedelgem, Belgium). Other chemicals are Prolabo products (VWR, Avantor, France) and they were used as received.

### 2.2. Synthesis Procedures

#### 2.2.1. Synthesis of Magnetite Nanoparticles

The so-called Massart method was used for the synthesis of Fe_3_O_4_ nanoparticles [65]. The hydrothermal co-precipitation procedure consists of mixing Fe(II) and Fe(III) salts (i.e., FeSO_4_·7H_2_O, 5.0 g and hydrated ammonium ferric sulfate (NH_4_)Fe(SO_4_)_2_·12H_2_O, 17.35 g) in 50 mL of demineralized water under vigorous stirring at 40–50 °C for one hour. Sodium hydroxide (5 M) was used for adjusting the pH to 10–12 and promoting the precipitation of the nanoparticles. The reaction was prolonged for five hours at 45 °C, under constant agitation. The precipitate was washed with Milli-Q water till neutralization of the rinsing bath; the nanoparticles were magnetically separated, washed with acetone before being dried at 50 °C for ten hours.

#### 2.2.2. Phosphorylation of Guar Gum

**Scheme 1:**
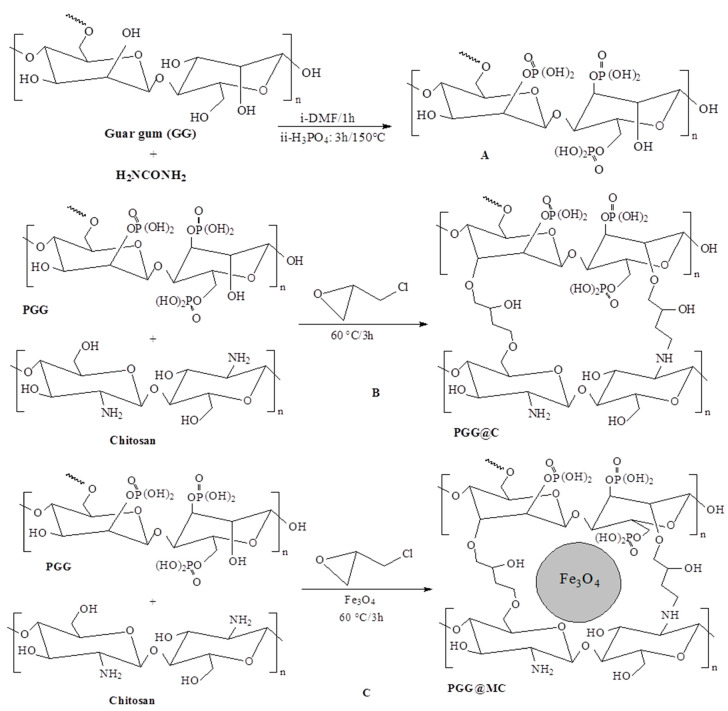
Pathways for the synthesis of sorbents (PGG@C and PGG@MC) ((**A**) PGG synthesis; (**B**) PGG@C synthesis; (**C**) PGG@MC synthesis).

Guar gum (1 g) was dissolved in DMF (55 mL before adding urea (15 g) under continuous agitation and until of the reagents were completely dissolved. The mixture was heated (under reflux) at 110 ± 5 °C for one hour. After cooling, phosphoric acid (13 mL, 85% *w*/*w*) mixed with DMF (20 mL), was dropped into the previous mixture and the temperature was risen to 150 °C for three hours. The precipitate was collected by vacuum filtration, washed with absolute ethanol before being air-dried to produce phosphorylated guar gum (PGG) as a dense white precipitation; the conversion yield reached 95%, in terms of mass balance, the amount of precipitate was close to 17 g (See Panel A in Scheme 1). 

#### 2.2.3. Synthesis of PGG-Chitosan Based Composites

A chitosan solution was prepared by dissolving the biopolymer (1 g) in an aqueous acetic acid solution (50 mL, 5% *w*/*w*). After complete dissolving, the PGG powder produced at the previous step was dropped into the solution and the mixture was heated under continuous stirring at 50 °C for one hour. Epichlorohydrin (2 mL) diluted in dioxane (5 mL) was dropped into the mixture and the pH was adjusted to 9 using NaOH solution (5 M) before heating the mixture to 60 °C for three hours. A pale brown precipitate (PGG@C, 5.1 g) is collected by filtration, washed with ethanol and finally dried (See Panel B in Scheme 1). The same procedure was used for preparing the magnetic composite (PGG@MC, see Panel C in Scheme 1), except that magnetite nanoparticles (3 g) were added simultaneously to PGG (in the first step of this procedure). The sorbent was collected as a black precipitate (8.5 g).

#### 2.2.4. Synthesis of Chit and MChit Composite

The biopolymer (chitosan, 1 g) was dissolved in aqueous acetic acid solution (30 mL, 5%, *w*/*w*). For the preparation of MChit, magnetite nanoparticles (3 g) were added to the chitosan solution under continuous stirring. The pH of the solution was controlled to 9 using NaOH (5 M), before maintaining the suspension under reflux for 1 h. The same method was applied to chitosan solution (without magnetite). The precipitates (pale yellow for Chit and black for MChit) were recovered by vacuum filtration (or magnetic separation), rinsed with water to remove unreacted reagents. The wet precipitates were dispersed into an alkaline solution (30 mL, pH 10) containing epichlorohydrin (2 mL); the mixture was maintained under agitation for 2 h at 50 °C. The precipitates were filtrated (Chit) or magnetically collected (MChit) before washing with water and ethanol and final air-drying at 50 °C for 10 h.

### 2.3. Characterization of Materials

The size and morphology of the sorbent were analyzed by transmission electron microscopy (TEM, JEOL 1010, JEOL Ltd., Tokyo, Japan). Morphological and chemical analysis studies were carried out using a scanning electron microscope coupled with an energy dispersive X-ray analyzer (XL30-ESEM, Philips, FEI, Thermo Fisher Scientific, Hillsboro, OR, USA). Fourier-transform infrared spectra were collected using a Mobile IR-Portable FT-IR (Bruker Optics, Billerica, MA, USA). The crystallinity of magnetite was characterized by X-ray diffraction patterns using an X-ray diffractometer X’Pert Pro (Philips, Eindhoven, The Netherlands). The textural analysis of materials was carried out on a high-speed surface area analyzer (Nova-e Series, Model 25, Quantachrome, Kingsville, TX, USA). The thermal degradation of the materials was studied using a TGA thermogravimetric analyzer (TGA 8000 Perkin Elmer, Villebon-sur-Yvette, France). X-ray diffraction patterns were acquired on a Malvern Panalytical Empyrean system (Malvern Instruments, Malvern, UK) equipped with an XPert MPD diffractometer, Generator: 30 mA and 40 kV for generator setting and CuK_α_ radiation.

### 2.4. Sorption Procedures

The sorption tests were performed in batch systems. A fixed volume of solution (V, L), containing a fixed metal concentration (C_0_, mmol L^−1^) at pH_0_ (initial pH value) was mixed with a given amount of sorbent (m, g) for t hours at room temperature (21 ± 1 °C). For kinetics, samples were collected, filtrated (and/or magnetically separated) at fixed contact times. The experimental conditions were adjusted in function of the specific experiments (the detailed experimental conditions are reported in the caption of the figures). The standard conditions correspond to C_0_: 100 mg U L^−1^ (~0.42 mmol U L^−1^); pH_0_: 4; sorbent dosage (m/V): 0.833 g L^−1^; time 24 h. The pH was not adjusted during the sorption step but the equilibrium pH (pH_eq_) was monitored at the end of the experiment. For sorption isotherms, the initial concentration varied between 0.042 and 2.05 mmol U L^−1^. Desorption was operated using 0.2 M HCl solutions; desorption kinetics were tested on metal-loaded sorbents collected from uptake kinetics, using the same procedure (SD: 2.52 g L^−1^; time: 2 h). For recycling tests, the same procedures were used with water rinsing steps between sorption and desorption operations. Selectivity tests were performed with equimolar multi-component solutions (C_0_: 0.5 mmol L^−1^), according to the same methodologies. 

The analysis of residual metal concentrations (C_eq_, mmol U L^−1^) was performed using the so-called Arsenazo III colorimetric method at λ: 655 nm for uranium [66] and 654 nm for rare earth elements [67]. In the case of mixtures, uranium concentration was analyzed by the oxidimetric titration method against ammonium vanadate [68,69]. Absorbance was measured using a Shimadzu 160A (Shimadzu Corporation, Kyoto, Japan). The analysis of other metals was performed by atomic absorption spectrometry (Unicam 969, Thermo Electron Corporation, Waltham, MA, USA). The sorption capacity (q, mmol U g^−1^) was calculated using the mass balance equation: q = (C_0_ − C_eq_) × V/m.

The modeling of uptake kinetics and sorption isotherms were performed using the conventional equations reported in Table S1a,b, respectively. The parameters were calculated using non-linear regression analysis (provided by Mathematica^®^ software, Wolfram Research, Champaign, IL, USA).

### 2.5. Antimicrobial Tests

The antibacterial activity of prepared composite (PGG@C and PGG@MC) was assessed using the agar well diffusion method against *Bacillus subtilis* ATCC6633, *Staphylococcus aureus* ATCC6538 as Gram+ bacteria, and *Escherichia coli* ATCC8739, *Pseudomonas aeruginosa* ATCC9022 as Gram− bacteria. Each bacterial species was individually streaked on Muller Hinton agar plate (ready prepared, Oxoid, Thermo Fisher Scentific, UK) using sterilized cotton swaps. Wells (0.8 mm in diameters) were prepared onto the inoculated plated before being filled with 100 µL of suspension (400 µg/1.0 mL DMSO). The plates were kept in the refrigerator (i.e., 4 °C) for 1 h, followed by incubation at 37 °C for 24 h. The results were recorded as the diameter of the zone of inhibition (ZOI) formed around each well by mm [70]. DMSO was running with the experiment as negative control. The ZOI was expressed as the mean ± standard deviation of three independent replicates. Data were subjected to analysis of variance (ANOVA) by the statistical package SPSS v17 (IBM, Armonk, NY, USA). The mean difference comparison between the treatments was analyzed by the Tukey HSD test at *p* < 0.05. 

## 3. Results and Discussion

### 3.1. Characterization of Materials

#### 3.1.1. Morphological Analysis-SEM and TEM

**Figure 1:**
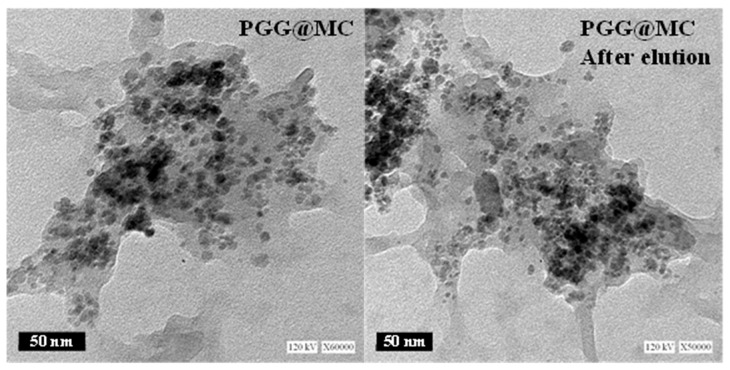
TEM images of PGG@MC (before and after sorption/desorption cycle, herein called: after elution).

The size of magnetite nanoparticles (magnetite NPs, dark objects) immobilized in the functionalized biopolymer matrix (light-colored) can be observed in Figure 1: dispersed particles predominate (with sizes ranging between ~5 nm and ~8 nm); although some aggregates or assemblies may be also observed. The distribution of the NPs is not homogeneous some zones of the composite are less densely filled. The same analysis was performed after a sorption/desorption cycle. The TEM observation does not show apparent changes in both the size and the distribution of the NPs in the composite after use. 

Figure S1 shows the morphology of sorbent particles (PGG@C and PGG@MC). The PGG@C particles are irregular in both size (from ~5 µm to 50–60 µm for the largest, consistently with the sieving of milled material at 63 µm) and shape (roughly cubic to ovoid objects, with irregular surfaces). On the opposite hand, the PGG@MC shows smaller objects (powdered structure) probably due to the effect of magnetite NPs.

#### 3.1.2. Textural Analysis-BET

The measurements of specific surface area (S_BET_, m^2^ g^−1^) and porous volume (V_p_, cm^3^ g^−1^) for PGG@C and PGG@MC clearly show the beneficial effect of the incorporation of magnetite NPs on the textural properties of the sorbent. Indeed, the textural parameters increase by one order of magnitude in the presence of magnetite NPs: S_BET_ increases from 5.06 m^2^ g^−1^ for PGG@C to 80 m^2^ g^−1^ for PGG@MC, while the porous volume reaches 26.4 cm^3^ g^−1^ for PGG@MC (against only 1.66 cm^3^ g^−1^ for PGG@C). 

#### 3.1.3. Structural Analysis-XRD

The presence of magnetite is attested by the magnetic separation of PGG@MC: Figure S2 shows the efficiency of a magnetic bar to separate sorbent microparticles from the solution. This figure also shows the XRD diffraction pattern of the sorbent, which is characterized by six peaks reported at 2*θ* = 30.33, 35.65, 43.27, 57.50, 63.08 and 74.41 degrees. These signals correspond to magnetite (JCPDS: 01-075-0449) [71,72]. However, the pattern is also very close to the pattern obtained for maghemite (JCPDS: 01-089-5892); this structure is favored, especially for nano-objects by the oxidation of the magnetite.

Though the 2*θ*-angles (and their relative intensities) for the sorbent are closer from the characteristics of magnetite, it is not possible rejecting that, at least, a fraction of the magnetic NPs could be oxidized and converted into maghemite [72]. The d-spacing values are also reported. For the most intense peak (at 2*θ* = 35.65 position), the d-spacing is 2.51830 Å. The application of the Scherrer equation allows approaching the average value of crystallite size [71], close to 8.7 nm, which is consistent with the order of magnitude of the NPs determined by TEM (Figure 1). 

#### 3.1.4. Thermogravimetric Analysis-TGA

**Figure 2:**
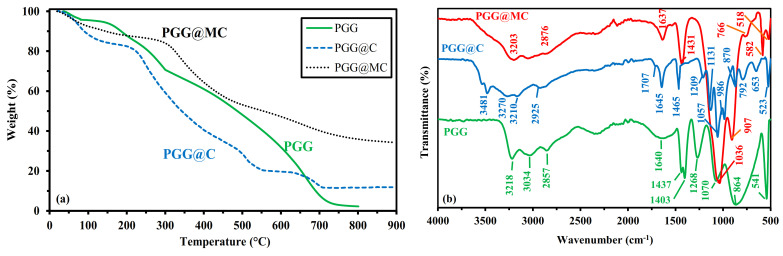
Characterization of PGG, PGG@C and PGG@MC materials: (**a**) TGA curves, (**b**) FTIR spectra.

The thermal degradation profiles are compared for PGG, PGG@C and PGG@MC in Figure 2a (and Figure S3). The TGA of PGG is characterized by four steps:

(a)  Below 151 °C, the weight loss reaches 6%, corresponding to water release and volatile matters.
(b)  Between 151 °C and 288.1 °C, the thermal degradation (about 60.5%) begins with probable dissociation of the linkages between phosphonate groups and GG. Singha et al. [50] reported the main degradation of GG between 230 °C and 320 °C (with a 49% weight loss), while the grafting of other functional groups extended the temperature range up to 400 °C.
(c)  Between 288.1 °C and ~600 °C, the degradation continues progressively and almost linearly (weight loss ~38%). This step in the process corresponds to the formation of the char resulting from backbone degradation.
(d)  Between ~600 °C and ~720 °C, a steeper step is observed corresponding to 28% weight loss, associated with char degradation.

It is interesting to note that despite the presence of phosphorus-based groups, which are well known for their fire-protecting properties, the composite loses about 98% of its weight at 800 °C: ashes represent less than 2%. This degradation profile is notably progressive with limited waves compared with the materials associated with chitosan (PGG@C and PGG@MC). Much higher residues at 800–900 °C also characterize the profiles of chitosan-based composites: 11.8% for PGG@C and up to 34.3% for PGG@MC. This difference between the two composites is directly correlated to the presence of stable magnetite, which proportion in the final product is close to 23%. This value is lower than the expected fraction of magnetite in the composite (based on the synthesis procedure: all other parameters maintained constant, the introduction of magnetite led to an increase in the amount of composite from 5.1 g to 8.5 g (for PGG@C and PGG@MC, respectively); this means a fraction of ~36%. The grafting of PGG on chitosan (PGG@C) increases the final stability of the composite, although (a) the degradation profile is shifted toward lower temperatures and (b) the transitions are more marked. 

In the case of PGG@C, several waves can be observed apart the initial water and volatile compounds release (below 193 °C–17.2% weight loss). The second phase corresponds to the temperature range between 193 °C and 500 °C (loss ~50%, which is followed by a steep weight loss, about ~10%); this may be associated with the degradation of linkages, carbohydrate backbones, and amine degradation from chitosan. The weight loss stabilizes up to ~630 °C, followed by a new degradation step up to 710 °C (about 9% weight loss): this is the final degradation of the char formed at temperature below 500–550 °C. The total weight loss reaches 88.15%. On the opposite hand, the incorporation of magnetite nanoparticles increases the thermal stability of the composite expressed both in terms of onset temperatures but also in the final residue (close to 34.3% at 900 °C), logically attributed to residual magnetite (after ultimate degradation of the organic char). For PGG@MC, the release of water and volatile organics represent about 10% at 145 °C, the weight loss progressively increases up to 283 °C (weight loss: ~5%), before detecting two degradation steps up to 485 °C (weight loss: ~31%) and up to 720 °C (weight loss: ~16%; char degradation). The little complementary weight loss (~4%) occurring at higher temperature may be associated to changes in magnetite phase. The total weight loss stands to 65.7%.

These differences between the three materials are confirmed by the DrTG curves (Figure S3). Several weak peaks are observed on PGG profile but the highest peak is detected at 651.6 °C. In the case of PGG@C, the highest peak appears at 508.0 °C, while a broad and poorly resolved band reaches a local extreme at 318.0 °C. The amplitude is increased with chitosan and even more with the magnetic composite. In the case of PGG@MC, the sharp maximum is detected at 473.9 °C, while a smaller broad band is identified at 253.3 °C. The grafting of PGG to chitosan affects the thermal degradation of the polymer composite, while the incorporation of magnetite nanoparticles decreases the temperature for intermediary degradation steps but globally increases the stability of the composite.

#### 3.1.5. Chemical Characterization–FTIR Spectroscopy

**Figure 3:**
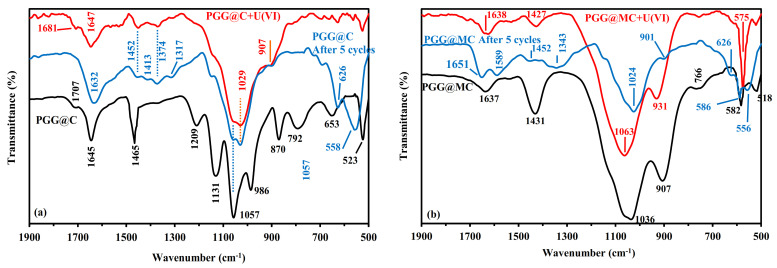
FTIR spectra of PGG@C (**a**) and PGG@MC (**b**), after U(VI) sorption and after 5 cycles of recycling (focus on Wn. Range: 1900–500 cm^−1^—Full Wn. Range see Figure S5).

Fourier-transform infrared spectroscopy can be used for characterizing the functional groups present on the sorbents (Figure 2b and Figure S4), but also for identifying those of the functional groups that are involved in metal binding (for supporting the interpretation of sorption mechanisms (Figure 3 and Figure S5). At the highest wavenumbers (Wn, in the range 3600–3200 cm^−1^), important differences are reported between PGG and chitosan-supported materials. In the range 3200–3300 cm^−1^, the OH stretching vibration is identified by a strong peak in PGG (GG naturally bears many hydroxyl groups), its relative intensity decreases with chitosan-supported material (because of the decrease in the relative proportion of OH-bearing GG). In the case of chitosan-bearing materials, a new peak appears at 3481 cm^−1^, which can be clearly assigned to NH stretching vibration. The zone 2950–2800 cm^−1^ corresponds to –CH stretching vibrations, with a marked predominance of the symmetric stretching vibration –CH_2_ for PGG (compared with other materials that show significant contributions of asymmetric stretching vibration at 2935 cm^−1^). A sharp peak band (well defined at 1645 cm^−1^) is appearing on the spectrum of PGG@C; this is usually assigned to amine (primary and/or secondary) stretching vibration [73], possibly overlapped with amide group (C=O stretching vibration, from the contribution of non-deacetylated chitosan) [74]. For PGG@MC a broader band is observed which may be explained by the convolution with the peak appearing at around 1707 cm^−1^ on PGG@C; this peak corresponds to C=O stretching in esters (correlated to the effect of epichlorohydrin crosslinking). The PGG spectrum is presenting a very broad and poorly resolved band centered around 1640 cm^−1^, which may include contributions of ring stretching at 1648–1642 cm^−1^ [75], –OH stretching (from phosphonate groups), water (as occurring for the regioselective phosphorylation of alginate, mediated by urea, [76]). The well-defined peak at 1645 cm^−1^ on PGG@C is also clearly identified on the spectrum of MChit, as a tracer of chitosan (and more generally carbohydrate). This convolution does not allow fine identification of the respective functional groups. Another peak is identified around 1465 cm^−1^ (identified on PGG@C as a single peak, on PGG as part of a double-peak at 1437 cm^−1^, and in PGG@MC as part of a broader peak, at 1480–1420 cm^−1^); this peak may be assigned to primary or secondary –OH bending vibration, overlapped with CH_2_ vibration in the case of PGG. In PGG spectrum, the peak at 1437 cm^−1^ includes CH_2_ deformation and bending vibrations [75]; this signal overlapped with the signal at 1465 cm^−1^ in PGG@MC probably due to the contribution of magnetite. For wavenumbers lower than 1300 cm^−1^, the differences between the three materials are more marked.

In the case of PGG, four peaks are identified:

(a) 1268 cm^−1^: P=O stretching in phosphonate.
(b) 1070 cm^−1^: primary –OH stretching vibration, with contribution of P-OH stretching vibration.
(c) 864 cm^−1^ (broad band): galactose and mannose rink (with possible contributions of (1–4) linkages at ~930 cm^−1^, and P-O stretching vibration at Wn > 900 cm^−1^ and P-O-C deformation vibration at Wn < 850 cm^−1^).
(d) 541 cm^−1^: P-O-C stretching vibration.

The synthesis of PGG@C and PGG@MC is strongly changing the FTIR profile due to the appearance of the fingerprint of chitosan carbohydrate ring that overlaps with the spectrum of GG. On PGG@C, strong peaks appear at 1209 cm^−1^, 1057 cm^−1^, 986 cm^−1^, and 870 cm^−1^, these peaks correspond to C-O stretching vibration in chitosan backbone [74]. This strong multiple-contribution overlaps with the peaks representative of GG and phosphonate moieties (and their relevant signals). The peak at 792 cm^−1^ is assigned to OH bending vibration. In the case of PGG@MC the typical peak observed at 582 cm^−1^ is a clear marker of magnetite [72,77]. This peak appears at 577 cm^−1^ for MChit and 565 cm^−1^ for MGG. The successive changes of FTIR spectra confirm the efficient phosphorylation of GG (PGG), the association with chitosan (PGG@C) and the incorporation of magnetite NPs (PGG@MC). 

#### 3.1.6. Surface Charge–pH_PZC_

Figure S6 shows the application of the pH-drift method for the determination of the pH_PZC_ values for PGG@C and PGG@MC, which are close to 5.60 and 4.84, respectively. This means that in acidic solutions the sorbents will be protonated with possible repulsion effects on the sorption of metal cations. The global positive charge progressively decreases with increasing the pH turning negative above the respective pH_PZC_ values. The sorbent is mainly constituted of chitosan, guar gum and phosphonate groups, which have specific acid-base properties. Hence, Sorlier et al. [78] reported that the pK_a_ values of the amine groups on chitosan depends on the degree of acetylation (DA) and the degree of neutralization of the biopolymer; however, for the most conventional commercial sample (with DA below 20%), the pK_a_, at full neutralization, stands between 6.3 and 6.4. On the other side, Pal et al. [79] reported the isoelectric point of GG close to 2.2: GG in acidic conditions may be considered neutral, while in mild acid conditions the biopolymer turns negatively charged. The first ionization constant of phosphonic acids depends on the side chain bound to phosphorus; however, for short chains the pK_a_ values are close to 2.4 [80]. It means that GG and phosphonate groups have similar acid behavior, while chitosan is bringing base properties (associated with amine groups). The different compounds and their interactions modulate the pH_PZC_ values of the two sorbents. The shape of the curves shows some breaks in the slopes that could precisely be related to the acid base contributions of the acid and base moieties (for example, around pH_0_ 6–6.5). Singha et al. [50] reported the influence of the composition of guar gum-based terpolymer on their pH_PZC_ values. These values were close to 5.85–6.07 in the case of the grafting of GG with acrylamide sodium propanoate (through solution polymerization of acrylamide (AM) and sodium acrylate (SA)) [81]. It is noteworthy that despite the coating of magnetite nanoparticles with the composite, the magnetic compartment affected the acid-base properties; provoking the slight shift of pH_PZC_ toward lower values (5.60→4.84). This shift is unexpected since the pH_PZC_ value of magnetite was reported in the range 6.8 [82] to 7.7 [83].

### 3.2. Sorption Performances–Synthetic Solutions

#### 3.2.1. Sorption Mechanisms Correlated with FTIR Analysis

The effect of U(VI) sorption on PGG@C FTIR spectrum is illustrated by Figure 3 and Figure S5. The figure also shows the FTIR spectrum of the sorbent after five recycling steps (meaning after the fifth sorption/desorption cycle). The width of the peak at 1645 cm^−1^ is enlarged after metal sorption, indicating that the chemical environment of amine groups is affected by U(VI) binding. It is noteworthy that the desorption of the metal (after the fifth cycle) does not fully restore the sharp peak initially observed at 1645 cm^−1^; this may be also due to the protonation of amine groups (after acidic desorption). The sharp peak at 1465 cm^−1^ is only strongly reduced while other peaks are appearing at 1452 cm^−1^ (shift of the initial peak) and at 1374 cm^−1^. The peak at 1209 cm^−1^, which was assigned to P=O asymmetric stretching vibration almost disappears; meaning that phosphonate groups contribute to U(VI) sorption. The series of peaks representative of chitosan carbohydrate backbone (1180–920 cm^−1^) is globally respected; although less resolved. However, the peak at 1057 cm^−1^ is split with appearance of a peak at 1029 cm^−1^ (which could be attributed to a chemical environment similar to P-OR esters). This band was also observed on the FTIR of uranyl nitrate (associated with sharp peaks at 940 cm^−1^ and 800 cm^−1^) [84]. The wavenumber of U-O stretching is affected by the complexation with phosphate in the range 950–916 cm^−1^ depending on the composition of the extraction media [85]. The convolution of signals in this area and the broadening of the band may explain the disappearance (by superposition) of the peak at 986 cm^−1^. The peaks at 870 cm^−1^ and 792 cm^−1^ (OH bending vibrations overlapped with C-O stretching of chitosan backbone) are also strongly reduced after metal sorption and the acid desorption does not allow restoring these peaks on FTIR spectra. With U(VI) binding, a significant shoulder can be detected at 907 cm^−1^; concomitantly with the peak at 1029 cm^−1^; this peak (shifted from 940 cm^−1^) may be considered a tracer of uranyl binding. The intensity of the peaks at 653 cm^−1^ and 523 cm^−1^ (tracers of phosphonate group) are significantly reduced and shifted toward lower (i.e., 628 cm^−1^) and higher (i.e., 563 cm^−1^) values, respectively. These observations mean that amine groups, hydroxyl groups, and phosphonate groups are involved in uranyl binding. It is noteworthy that for many of these bands the reuse and desorption of uranyl does not restore the FTIR spectrum. However, the comparison of sorption and desorption efficiencies investigated over five cycles shows a remarkable stability in performances (see below); this means that the apparent chemical changes are not breaking at all in terms of metal binding. The analysis of the FTIR spectra for PGG@MC (before, after U(VI) binding and after fives cycles of sorption and desorption) are roughly confirming the same trends, pending the specific modifications of the spectra associated with the interactions of the matrix with magnetite nanoparticles (less–resolved spectrum in the fingerprint area of chitosan carbohydrate ring. The most significant changes are observed on the vibrations associated to amine groups (in the region around 1637 cm^−1^), OH bending groups (i.e., around 1431 cm^−1^), phosphonate-based vibrations (shifts 1036 cm^−1^ to 1063 cm^−1^), and obviously the appearance of the peak at 931 cm^−1^ (representative of uranyl U-O stretching in UO_2_^2+^) [85].

#### 3.2.2. PH Effect

**Figure 4:**
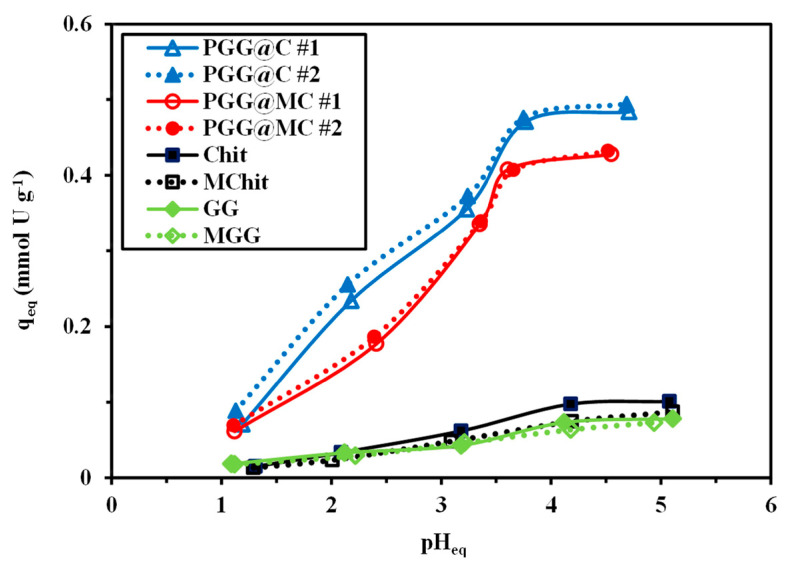
Effect of pH_eq_ on the sorption of U(VI) using PGG@C and PGG@MC (and intermediary products: Chit, MChit, GG, and MGG) (C_0_: 0.42 mmol U L^−1^; Sorbent dosage, SD: 833 mg L^−1^; Time: 48 h; v: 200 rpm; T: 21 ± 1 °C).

The pH influences the overall charge of the sorbent (protonation/deprotonation of reactive groups), but also the speciation of metal ions in the solution (Figure S7). Therefore, this parameter strongly controls the sorption performance. Figure 4 shows the progressive increase of U(VI) sorption capacity with incrementing the equilibrium pH (pH_eq_), especially between pH 1 and pH 4; above pH 4.2 the sorption capacity stabilizes for conventional materials; the stabilization begins around pH 3.7 for phosphorylated sorbents. The sorption was not tested at pH_0_ above 5 because of the occurrence of precipitation phenomena (preceded by the formation of polynuclear species and colloidal species). This figure also illustrates the beneficial effect of the phosphorylation of the materials. Indeed, for Chit and GG (and their magnetic composites) the sorption capacity (under selected experimental conditions) increases from ~0.02 mmol U g^−1^ to 0.06–0.1 mmol U g^−1^ from pH_0_ 1 to 4.5. On the opposite hand, the phosphorylated materials show sorption capacities increasing from ~0.07 mmol U g^−1^ to 0.43–0.49 mmol U g^−1^. The functionalized composites showed sorption capacities increased by 4 to 5 times; this is a clear justification of the strategy adopted for designing the sorbent. The incorporation of magnetite leads to a decrease in sorption capacity by ~13%. This decrease can be partially explained by the decrease in the effective amount of reactive groups; indeed, TGA tests showed that about 23% of the mass of the sorbent is constituted of magnetite with lower reactivity for U(VI) than the phosphorylated polymer.

The quasi-linear increase in the sorption capacity with the pH (below pH_eq_ 4, which is below the pH_PZC_ of both PGG@C and PGG@MC) is directly correlated with the progressive deprotonation of reactive groups at the surface of the sorbent. Figure S7 shows the large predominance of free UO_2_^2+^ species at pH below 4; with progressive appearance of hydrolyzed and polynuclear species at pH 3.5. All the species present in solution are cationic causing electrostatic repulsion with protonated reactive groups; increasing the pH progressively reduces this repulsion: the sorption capacity increases. The stabilization at pH superior to 3.7 for PGG@C and PGG@MC may be correlated with the appearance of polynuclear species. In the case of uranyl sorption on fungal biomass, the most reactive groups are amine groups of chitosan-based constituent of the cell wall; Guibal et al. [86] showed the preference of these amine groups for polynuclear uranyl species. In the present case, the amine groups of chitosan may contribute to uranyl binding; however, phosphonate appear to be more reactive and to have marked preference for free-uranyl species because of the effects of steric hindrance and differential affinity. 

The sorption process is frequently associated with pH changes that may be caused by proton binding or release on the sorbent and/or U(VI) equilibrium displacement due to metal binding. Figure S8 shows the pH variation occurring during metal sorption. At pH below 3.5, the equilibrium pH tends to slightly increase (by less than 0.5 pH unit) for all the sorbents. Above pH 3.5, the pH is almost not affected by metal sorption in the case of Chit, MChit, GG and MGG while for phosphorylated sorbents the pH tends to decrease by 0.5 pH unit. The formation and partial binding of hydrolyzed species (which shifts uranyl speciation) may explain this pH decrease. The distribution ratio (D = q_eq_/C_eq_, L g^−1^) increases with equilibrium pH (Figure S9); its log_10_ plot may be used, in ion-exchange processes, for evaluating the stoichiometric exchange ratio between metal ions and protons through the determination of the slope. In the case of PGG@C and PGG@MC, the slope is closed to +0.5, meaning that two protons may be exchanged with one uranyl ion (Figure S9). In the case of Chit and MChit, the slope is close to +0.23–0.24, while for GG and MGG the slope tends to +0.17 (not shown). The phosphonate reactive groups involve different modes of interaction with uranyl than the raw biopolymers.

#### 3.2.3. Uptake Kinetics

##### Mechanical Agitation

**Figure 5:**
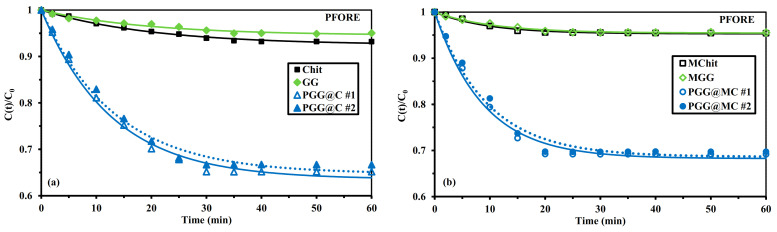
U(VI) uptake kinetics using Chit, GG, and PGG@C sorbents: non-magnetic (**a**) and magnetic (**b**) sorbents–Modeling with the PFORE (C_0_: 0.42 mmol U L^−1^; pH_0_: 4; Sorbent dosage, SD: 300 mg L^−1^; v: 200 rpm; T: 21 ± 1 °C).

Under selected experimental conditions, the uptake kinetics is relatively fast: about 50 min for raw Chit and GG polymers and 30 min for phosphorylated materials (Figure 5). It is noteworthy that the incorporation of magnetite nanoparticles allows reducing the equilibrium time to 40 min for raw polymers and 20 min for PGG@MC. This beneficial effect of magnetite NPs counterbalances the slight reduction in sorption capacities at equilibrium (Table 1). 

Uptake kinetics may be controlled by different mechanisms, including resistances to bulk, external film and intraparticle diffusions, and the proper reaction kinetics (which may be fitted by models such as pseudo-first and pseudo-second order rate equations, PFORE and PSORE, respectively). Providing a sufficient agitation allows reducing the contribution of resistance to bulk and film diffusion (mainly active within the first minutes of contact). The three models of resistance to intraparticle diffusion (the so-called Crank equation, [87]), PFORE and PSORE are reminded in Table S1a [88].

Table 1 summarizes the parameters for the PFORE, while the parameters for PSORE and RIDE are reported in Tables S2 and S3. The comparisons of determination coefficients and values of Akaike information criterion (AIC) confirm that the PFORE fits experimental profiles much better than the PSORE (Figure S10 and Table S2) and the RIDE (Figure S11 and Table S3). 

The discussion of mathematical fits may introduce some interpretation bias. Simonin [89] and Hubbe et al. [90] identified and discussed some of the major errors that may lead to misinterpretation of the kinetic profiles based on inappropriate selection of experimental conditions, such as, among others, excessive variation of solute concentration (i.e., excessive sorbent compared with solute concentration). Concluding that the mathematical fit of experimental profile can explain and differentiate between, for example, physical and chemical sorption may be erroneous. Hubbe et al. [90] concluded more specifically that frequently the experimental conditions do not fulfill the assumptions associated with the PSORE, making the interpretation debatable. 

The calculated values for q_m,1_ (in the PFORE) slightly overestimate the effective sorption capacities at equilibrium by less than 4% for phosphorylated sorbents. As expected, the equilibrium sorption capacities for phosphorylated sorbents are about 4 to 5 times higher than the values obtained for raw polymers (both non-magnetic and magnetic samples). It is noteworthy that the experimental profiles (and their modeling) are reproducible (variations remain below 2%). The apparent reaction rates (i.e., k_1_) are also remarkably reproducible (variation in the range 3.8–7.6%) with higher value for PGG@MC than for PGG@C: (faster for magnetic material, consistently with equilibrium time): 6.69 ± 0.15 × 10^−2^ min^−1^ and 10.9 ± 0.4 × 10^−2^ min^−1^, respectively. These values are higher than those obtained with Chit and GG sorbents; the incorporation of magnetic NPs also increases the value of the apparent rate coefficients for the composite biopolymers (almost doubled). This may be directly connected by the improvement of textural properties of the sorbent with the incorporation of magnetite nanoparticles (both in terms of S_BET_ and V_p_, see Section 3.1.2). The phosphorylation improves not only the sorption capacity (through the increase in the density and reactivity of functional groups) but also the mass transfer properties (higher density at the surface of the sorbent). 

Though the RIDE fails to fit the experimental profile in the intermediary time range (5–45 min), the model can be used for approaching the order of magnitude of the intraparticle diffusion coefficient and comparing the different sorbents. Awakura et al. [91] reported the decrease of uranyl diffusivity coefficient (from 5 × 10^−8^ m^2^ min^−1^ to 3 × 10^−8^ m^2^ min^−1^) with increasing the concentration of sulfate in the solution containing high concentrations of sulfate (i.e., 100→200 g L^−1^). Taking into account the size of sorbent particles (below 63 µm) and the kinetic profiles modeled with the RIDE, the effective diffusivity coefficient in the sorbents are about 4 orders of magnitude lower: 1.16–1.33 × 10^−11^ m^2^ min^−1^ for non-magnetic sorbents and 4.4–5.3 × 10^−12^ m^2^ min^−1^ for magnetic sorbents (being comparable for raw biopolymers and functionalized materials). This gap clearly demonstrates that the resistance to intraparticle diffusion plays a non-negligible role in the control of mass transfer.

##### Sonication-Assisted Sorption

The mass transfer properties may be enhanced using different strategies such as playing with the porosity of the materials, and decreasing the size of sorbent particles. However, recent studies have clearly showed the beneficial effect using alternative modes of agitation. The sorption performance can be strongly improved by application of ultrasonic treatment (UT) [92] or microwave activation [93]); in the latter case the kinetic rate is drastically accelerated while the equilibrium sorption is dramatically decreased.

**Figure 6:**
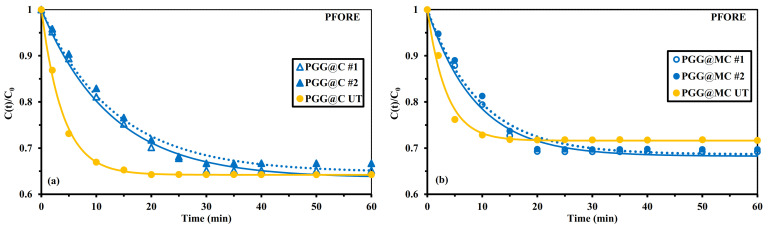
Effect of ultrasonic treatment (UT) on U(VI) uptake kinetics using Chit, GG, and PGG@C sorbents: non-magnetic (**a**) and magnetic (**b**) materials–Modeling with the PFORE (C_0_: 0.42 mmol U L^−1^; pH_0_: 4; Sorbent dosage, SD: 300 mg L^−1^; v: 200 rpm; T: 21 ± 1 °C).

Figure 6 confirms the enhancement of mass transfer properties when applying ultrasonic treatment (power: 160 W; frequency: 25 kHz): the equilibrium time decreases to 15 min (more than 98% of total sorption occurs within the first 10 min) and the initial slope of the kinetic profile is strongly increased. The apparent rate coefficients are significantly improved while using UT: from 7.69 ± 0.15 × 10^−2^ min^−1^ to 25.6 × 10^−2^ min^−1^ in the case of PGG@C and from 10.9 ± 0.4 × 10^−2^ min^−1^ to 28.7 × 10^−2^ min^−1^ in the case of PGG@MC. However, it is important reporting that the ultrasonic treatment hardly affects the sorption capacity at equilibrium for PGG@C (∆: −2%) while the sorption capacity decreases by 16% for PGG@MC. The ultrasonic activation is not really justified for the magnetic sorbent. It is noteworthy that the UT improves the fitting of experimental profiles by the PFORE. Figures S12 and S13 reports the modeling of kinetic profiles with the PSORE and the RIDE, respectively. The enhancement of mass transfer is also confirmed by the increase in the effective diffusivity (by a factor 2.4–2.8) when the system was submitted to UT (Table 1).

#### 3.2.4. Sorption Isotherms

**Figure 7:**
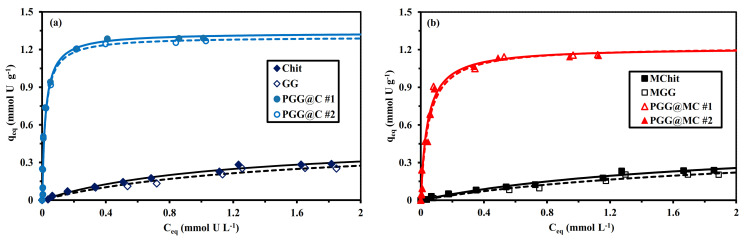
U(VI) sorption isotherms using Chit, GG and PGG@C sorbents: non-magnetic (**a**) and magnetic (**b**) materials–Langmuir modeling (pH_0_: 4; SD: 833 mg L^−1^; Time: 48 h; T: 21 ± 1 °C; v: 200 rpm).

The sorption isotherms represent the distribution of the solute between the liquid and solid phases for different metal concentrations, at fixed temperatures. The sorption isotherms for PGG@C and PGG@MC show similar trends (Figure 7): the sorption capacity steeply increases at low metal concentration (almost irreversible shape) followed by a saturation plateau reached for residual concentrations close to 0.4–0.45 mmol U L^−1^, and maximum sorption capacities close to 1.28 mmol U g^−1^ and 1.16 mmol U g^−1^ for PGG@C and PGG@MC, respectively. The loss in sorption capacity (i.e., 9.4%) is lower than expected, based on the actual fraction of magnetite in the sorbent (about 23%); this means that the presence of magnetite increases the accessibility and availability of reactive groups in the composite. 

The sorption isotherms of raw biopolymers (and their magnetic composites) shows much less favorable sorption isotherms: lower initial slope (assimilated to sorbent affinity for the metal) and lower sorption capacities. The saturation plateau is probably not reached even for residual concentrations as high as 2 mmol U L^−1^. The sorption isotherms have been modeled using some of the most conventional models (i.e., Langmuir, Freundlich and Sips equations, Table S1b). The shape of the sorption isotherms for phosphorylated sorbents (with a saturation plateau) is not consistent with the power-like function adopted by the Freundlich equation. The Langmuir model supposes the sorption to occur as a monolayer with no interactions between sorbed molecules and with homogeneous distribution of sorption energies at the surface of the sorbent. The Langmuir equation is more appropriate for approaching the saturation plateau of the experimental sorption isotherms. This is confirmed by the comparison of the determination coefficients and the AIC values (Table 2, Table S4 and Table S5). 

The calculated values of q_m,L_ overestimates the experimental sorption capacities at saturation of the sorbent (by 2.5% to 5.6%). The affinity coefficient (b_L_) is about 5 times greater for functionalized sorbents compared with raw biopolymers (both on non-magnetic and magnetic forms). It is remarkable that the affinity coefficient decreases from 47.1 ± 0.5 L mmol^−1^ to 22.0 ± 1.7 L mmol^−1^ after the incorporation of magnetite NPs. The product q_m,L_ × b_L_ (L g^−1^) is analogous to a distribution coefficient; this parameter is correlated with the initial slope of the sorption isotherm. The highest the parameter, the greater the affinity of the sorbent for the target metal ion. These values clearly demonstrate the superiority scale: PGG@C >> PGG@MC >>> Chit > GG > MChit > MGG. The introduction of a third-adjustable parameter in the Sips equation does not significantly the quality of the fits of experimental parameters (as confirmed by the values of the AIC). The values of this supplementary parameter (i.e., n_S_) vary between 0.80 and 0.94 for PGG@C, while PGG@MC (between 1 and 1.2 for raw biopolymers and their magnetic composites); this confirms the weak impact of the Freundlich-type exponent in this equation for simulating the sorption isotherms.

Table S6 summarizes U(VI) sorption performances of a series of sorbents recently reported in literature (including commercial resins). These sorption properties are strongly controlled by experimental parameters and more specifically the pH of the solution. This makes the direct comparison of the performances relatively complex. Some materials shows outstanding sorption performances: for example, diethylenetriamine tethered mesoporous silica shows maximum sorption capacities as high as 3.87 mmol U g^−1^ at pH 6 [94]; this high pH value makes questionable the occurrence or contribution of precipitation phenomena (depending on the effective composition of the solution). Uranyl sorption reaches 2.63 mmol U g^−1^ for phosphonic acid functionalized cellulose at pH 5 [95]. On the opposite hand, some commercial resins show much lower sorption capacities applied for the treatment of very acidic solutions. Sorption capacity does not exceed ~0.3 mmol U g^−1^ for Tulsion CH-96 in 4 M HNO_3_ solutions [96], 0.49 mmol U g^−1^ for Purolite A400 at pH 1.9 [22], or 0.71 mmol U g^−1^ on magnetic phosphine oxide/amino functionalized sorbent at pH 0.5 [97]. At the pH selected for the current study (i.e., pH 4), the sorption performance for PGG@C and PGG@MC (i.e., 1.15–1.28 mmol U g^−1^ and 22–47 L mmol^−1^) is consistent with the highest values reported in terms of maximum sorption capacity and affinity coefficient for the most efficient alternative sorbents. The equilibrium time also corresponds to the values reported for the most efficient sorbents in terms of kinetic criteria. The functionalization of guar gum and its association with chitosan make the sorbents very promising for uranyl recovery. The next sections examine some issues on the selectivity and recycling possibilities of these materials for evaluation of their operative potential. 

#### 3.2.5. Sorption in Multi-Component Solutions–Selectivity

**Figure 8:**
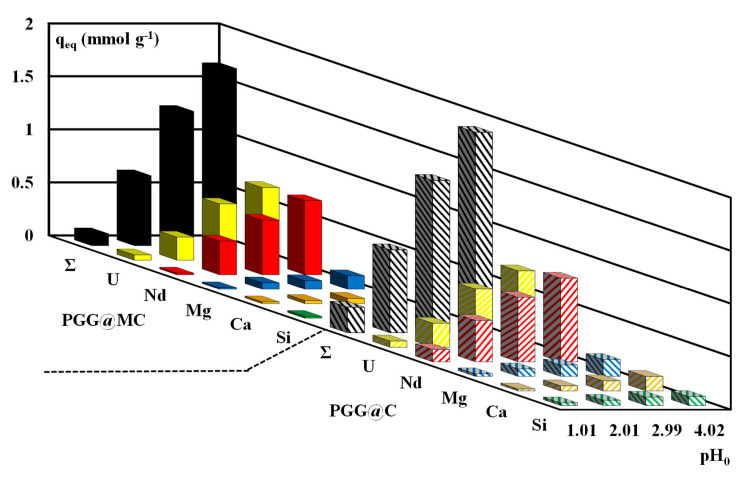
Effect of pH_0_ on the sorption capacity of PGG@C and PGG@MC for U(VI), Nd(III), Mg(II), Ca(II) and Si(IV) from equimolar multi-component solutions (C_0_: 0.5 mmol L^−1^; SD: 833 mg L^−1^; v: 200 rpm; T: 21 ± 1 °C; Time: 10 h; Σ: cumulative sorption capacity).

The effect of pH_0_ on the sorption of U(VI) from multi-elemental equimolar solutions (i.e., U, Nd as representative of rare earth elements, REEs, Mg, Ca and Si as representative of aluminosilicate ore background) (Figure 8). This figure clearly demonstrates the marked preference of the sorbent for U(VI) and Nd(III) compared with Ca(II), Mg(II) and Si(IV), and confirms the enhanced sorption at pH close to 4. The sorption capacities of the PGG@MC sorbent reach ~0.8 mmol g^−1^ for U(VI) and Nd(III), while for the other metals (or metalloid) the sorption capacity does not exceed 0.16 mmol g^−1^ at pH_0_ 4. The sorbents have comparable sorption levels, under selected conditions, for U(VI) and Nd(III) The Pearson’s rules (hard and soft acid base theory) set that the hard acids react preferentially with hard bases (and reciprocally). The selected metals (including alkali earth metals and Si(IV)) are all classified as hard bases. The functional groups on the sorbents (i.e.; amine and phosphonate groups) are considered hard bases; this may explain the affinity of the sorbent for U(VI) and Nd(III) but does not support the relatively weak sorption of other competitor ions. The comparisons of hydrated radius [98], ionic radius and softness [99], solution-phase electronegativity [100] for the different elements do not allow correlating these physicochemical properties with their sorption onto PGG@C and PGG@MC.

It is noteworthy that the cumulative sorption capacities are close to 1.67 mmol g^−1^ and 1.89 mmol g^−1^ for PGG@C and PGG@MC; these values are higher (by 44–47%) than the maximum sorption capacities reported in Table 2 for U(VI) alone. This is a clear evidence that the different metals are not necessarily bound on the same reactive groups (nor the same mechanisms).

Selected experimental conditions (especially equimolarity) allows calculating the selectivity coefficient, *SC_metal1/metal_*_2_ according to:

(1) $$SC_{Me1/Me}=\frac{D_{Me1}}{D_{Me2}}=\;\frac{q_{eq,Me1}\times \;C_{eq,Me2}}{C_{eq,Me1}\times \;q_{eq,Me2}}$$

The *SC* values for U(VI) and Nd(III) against other metals for the different values of equilibrium pH are reported in Figure S14. The high affinity of the sorbents for both U(VI) and Nd(III) may explain the small values of *SC_U/Nd_* (and *SC_Nd/U_*) that range between 0.3 and 3. It is noteworthy that PGG@C has a little preference for Nd(III) against U(VI) at pH_eq_ 1.29 (SC_Nd/U_~2.1) contrary to PGG@MC that shows greater preference in acidic solutions for U(VI) over Nd(III) (SC_U/Nd_~3.3). Apart this specific issue, the two sorbents have roughly the same selectivity against other competitor ions. The selectivity for these strategic metals against alkali-earth metals and Si(IV) significantly increases with the pH:SC values range between 10 and 40 at pH_eq_~3.5. The scale of overall selectivity of the sorbents follows the trend: U(VI)~Nd(III) >> Mg(II) > Ca(II) >> Si(IV).

This scale cannot be directly correlated with the physicochemical properties of these metal ions (and metalloid), as reported above. This may result from the occurrence of different mechanisms and contributions of different reactive groups, in addition to the proper effects of metal speciation.

Actually, the pH_PZC_ of the two sorbents are 5.60 and 4.84 for PGG@C and PGG@MC, respectively. This means that the two sorbents are positively charged in the experimental range of pH variation (i.e., from 1 to 4). Merdivan et al. [101] investigated the sorption properties of a phosphonate-grafted resin for the removal of U(VI) and other transitions metal ions. They reported the highest selectivity of the phosphonate-resin for U(VI) against other heavy metals at pH 4. Obviously, increasing the pH progressively decreases the overall charge making it possible enhancing metal sorption. As observed for mono-component solutions, the sorption capacities increase with the pH for multi-metal compositions. It is noteworthy that for both magnetic and non-magnetic sorbents, the sorption capacities more specifically increase when the pH is shifted from 3 to 4. This may be associated to the dissociation of functional groups: the pK_a_ of primary amine groups of chitosan is close to 6.3, while phosphonate moieties have more acidic pK_a_ values. These are the last functional groups who are the most sensitive to pH (in the investigated range). 

Lanthanides, as do actinides, form a so-called inner transition series, characterized by strong ionic bonding and weaker covalent bonding properties. The deprotonation of phosphonate is thus expected to improve their sorption properties. According Xu et al. [102], who correlated the acid softness index with the Gibbs free energies of formation, actinides and lanthanides have stronger hard behavior than alkali-earth elements such as Ca and Mg. The selectivity scale follows this trend. Yang and Alexandratos [103] discussed the effect of several parameters such as the acidity of the solution on metal speciation and ligand protonation for the selective sorption of lanthanide on different resins in relation with the polarizability of the donor atoms, and heir hydration behavior. Combining appropriate acid-base properties, with hydrophilic reactive groups (for example grafting sulfonic groups) allow better matching the sorption to target metals (taking into account their softness, hydration and acid-base properties).

#### 3.2.6. Uranium Desorption and Sorbent Recycling

Different types of eluents can be used for the elution of uranium from metal-loaded resins and sorbents [104], including acid solutions [105] but also carbonate eluents [106,107,108]. Based on the sensitivity of sorption performance to pH, logically acidic solutions have been selected for processing the desorption of U(VI) from loaded sorbents. Hydrochloric acid solutions (0.2 M) have been used for processing the desorption of U(VI) from metal-loaded sorbents (collected from sorption kinetics): Figure S15 compares the kinetic profiles for the different materials. These curves confirm the high efficiency of 0.2 M HCl solution for the recovery of uranium: uranyl ions are completely eluted after 15–30 min. It is noteworthy that the incorporation of magnetite tends to enhance the velocity of elution: the curves are shifted toward lower contact times (by ~10 min). The desorption process is faster than the sorption one (equilibrium reached after 60 min).

The recycling of the sorbent is another important criterion for evaluating the potential for industrial application of newly designed sorbents. Table 3 compares the sorption and desorption performances for the two sorbents over five successive cycles. The sorption efficiency slightly decreases with the recycling; however, the loss at the fifth cycle does not exceed 5–6%. On the other side, the desorption is remarkably stable all along the five cycles: the desorption efficiency remains higher than 99%. These results show the high effectiveness of PGG@C and PGG@MC for sorbent recycling.

### 3.3. Treatment of Ore Leachate

#### 3.3.1. Ore Origin and Pre-Treatment

Uraniferous shale is found mainly in the Um Bogma Formation in the southwestern Sinai. The ore material was collected from Allouga mining area; Figure S16 shows the location and geological map of the relevant area. This ore material is characterized by a relatively high chloride concentration that, in most cases, ranges between 3–7% (*w*/*w*) (Table S7) [109]. Actually, the sedimentary rocks in Sinai represent a kind of exception in the field of uranium resources, regarding the levels of chloride. Chloride ions can be also identified in the pyro metallurgical treatment of uranium ores (roasting in the presence of Cl-salts). These high levels yield an adverse effect on uranium recovery from acid leachates [110]. Indeed, when the concentration exceeds 3 g L^−1^, chloride ions strongly compete with uranium for binding on quaternary amine resins (the type of resin commonly used for extraction of U from acid leachates). To avoid these problems, ore materials is water-washed prior to processing the leaching step. Actually, the duration of the washing process depends on the ore grade (chloride content), grain size and the flow rate of the washing water. The washing step was stopped when the outlet solution contained around 3 g Cl L^−1^. Figure S17 shows the evolution of the concentrations of Cl^−^ (g L^−1^) and U(VI) (mg L^−1^) for successive 20-mL volume fractions during the washing step of the ore. It is necessary processing 20 fractions (i.e., a total volume of 400 mL) to reach the target value (i.e., 3 g Cl L^−1^) for residual chloride ions in the washing pregnant solution (WPS). The effluent of this washing step (WPS, washing pregnant solution) was collected; the presence of chloride in the ore allowed extracting several metal ions, which make the effluent both highly contaminant and an environmental challenge for the operator. Table S8 reports the composition of the solution produced by water washing (volume: 400 mL) of 1 kg of ore (size: 7–15 mm), packed in a column (diameter: 9 cm; height: 90 cm). The column was fed down flow (2 mL min^−1^; i.e., superficial flow velocity, SFV: 0.02 m h^−1^). Relevant figures are the chloride content close to 34.6 g Cl L^−1^ and the concentration of uranium set at 9.05 mg U L^−1^. The initial pH of this solution is 6.3.

#### 3.3.2. Treatment of WPS (Washing Pregnant Solution)

The sorption tests were performed using the same batch procedure, testing three different values of pH: the natural pH (i.e., pH_0_ 6.3 and pH_eq_: 5.88–5.94), pH_0_ 2.01 (pH_eq_: 2.29–2.31), and pH_0_ 4.02 (pH_eq_: 3.85–3.98). After 5 h of contact, the residual concentrations of a series of metal ions (and Si(IV)) were measured. Figure S18 shows that sorption efficiency increases with equilibrium pH whatever the element considered. It is noteworthy that U(VI) uptake ranges between 83% and 89% for PGG@MC and PGG@C, respectively: the high affinity of the sorbents for U(VI) and the low initial concentration of uranyl (i.e., 38 µmol U L^−1^) can both explain this remarkable performance. For low-concentration metal ions (i.e., Zn(II) and Ni(II), 86 µmol Zn L^−1^ and 35 µmol Ni L^−1^) the sorbents have higher sorption efficiency for Zn(II) than for Ni(II) (both of them being part of the borderline class according Pearson’s rules [111]). Among the predominant metal ions (i.e., Al(III) and Ca(II) at 3377 µmol Al L^−1^ and 3590 µmol Ca L^−1^), only Ca(II) is significantly sorbed: efficiency ranges between 26% and 29% (less than 16%) at pH_eq_ ~5.9. For the elements having an intermediary concentration (i.e., 350–1240 µmol L^−1^), except for Mn(II) (efficiency ranging between 13% and 17%) the other metals ions (i.e., Cu(II) and Fe(III)) and Si(IV) are sorbed at levels between 27% and 39%.

**Figure 9:**
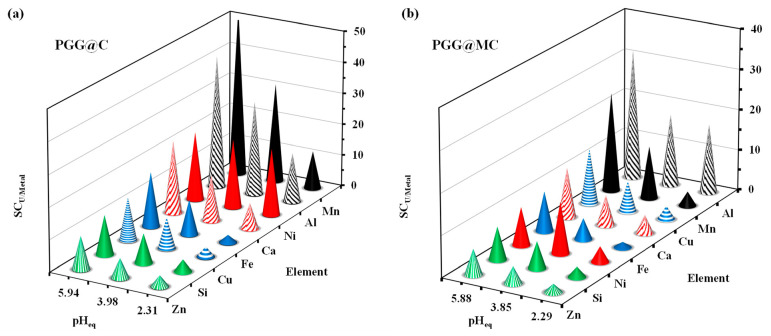
Selectivity coefficients for U against other elements present in the WPS for different pH_eq_ values using PGG@C (**a**) and PGG@MC (**b**).

The affinity of the sorbents for selected metals in such a complex solution may be also compared using the selectivity coefficient (SC_U/metal_). Figure 9 shows that the selectivity of the sorbents for U(VI) against other metal ions and metalloid increases with the pH (except for Ni(II) and PGG@MC where the selectivity for U(VI) over (Ni(II) reaches a maximum at pH_eq_ = 3.85). In addition, the values for PGG@C are generally greater than for PGG@MC. The selectivity scales are as follows:

For PGG@C: Mn(II) > Al(III) > Ni(II) > Ca(II) > Fe(III) > Cu(II) > Si(IV) > Zn(II).

For PGG@MC: Al(III) > Mn(II) > Cu(II) > Ca(II) > Fe(III) > Ni(II) > Si(IV) > Zn(II).

These scales cannot be directly correlated to the physicochemical properties of these metals ions (and metalloid), as reported in Table S9. It is also important reporting that U(VI) and most of the elements belong to the so-called Pearson’s hard class (hard acids), except Ni(II), Cu(II) and Zn(II), which are borderline. While Cu(II) and Ni(II) are in the middle of the scale, Zn(II) is the metal ions for which the selectivity coefficient is the lower. Predicting the preferences through the physicochemical characteristics of the metal ions is thus difficult. The differences in the concentrations, the coexistence of different reactive groups and the effects of metal speciation may explain the difficulty in correlating these data. Anyway, the optimum pH (corresponding to the natural pH) the lowest selectivity coefficients are close to 6 for PGG@C and up to 10 for PGG@MC for U(VI) against Zn(II). 

Figure S19 shows the semi-quantitative EDX analysis of the sorbents after being exposed to the WPS. As expected the major elements are associated with the sorbents: C, O, N and P for PGG@C, completed with Fe for PGG@MC (for magnetite). In addition, U element represents a substantial fraction of the sorbents (i.e., ~10% for PGG@C and ~6.6% for PGG@MC, *w/w*). Other metal ions are identified consistently with the trends reported above, and their initial concentrations: high amounts of Al, Ca, Si, in addition to Na, Mg.

Despite the huge amount of chloride in the solution (i.e., ~30 g Cl L^−1^), PGG@C and PGG@MC have outstanding sorption efficiency for U(VI) and remarkable selectivity against other metals (including those that are in excess).

### 3.4. Antibacterial Application

**Figure 10:**
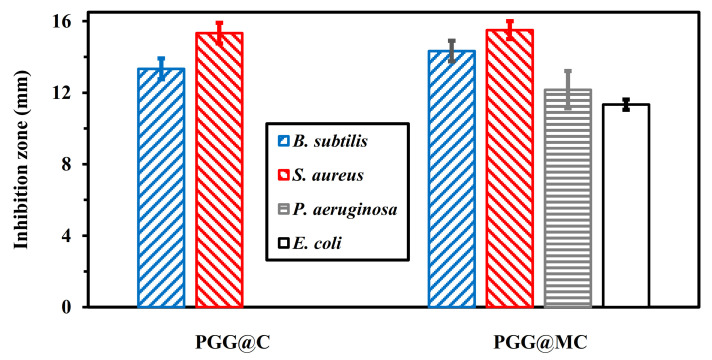
Testing of the antibacterial effect of PGG@C and PGG@MC using the exclusion zone method (note: no exclusion zone for PGG@C in the case of *P. aeruginosa* and *E. coli* strains).

The blank test showed that DMSO does not exhibit any activity against tested bacterial strains. Apparently, the antibacterial activities associated with PGG@MC composite are little higher than those observed with PGG@C (Figure 10): the ZOI reached 13.3 ± 0.5 mm and 14.3 ± 0.6 mm, respectively, for *B. subtilis,* and 15.3 ± 0.6 mm and 15.5 ± 0.5 mm for *S. aureus,* respectively. It is noteworthy that PGG@C does not exhibit any activity against Gram-negative bacteria as compared with PGG@MC. The ZOI caused by PGG@MC against *E. coli* and *P. aeruginosa* was 11.3 ± 0.3 and 12.2 ± 1.04 mm, respectively. However, the analysis of variance shows that the differences are not statistically significant between PGG@C and PGG@MC for Gram-positive bacteria (with *p* = 0.101), while there are statistically significant for Gram-negative bacteria (with *p* ≤ 0.001). These data are consistent with Haldorai et al. [61], who reported that the antibacterial activity of chitosan-doped magnetite (Fe_3_O_4_) was better than the activity of each one used alone. Magnetite/chitosan composite exhibited antibacterial activity against *E. coli* and *B. subtilis* with ZOI of 16.0 ± 0.5 and 18.0 ± 0.5 mm, respectively [70]. In their study on the inhibition of biofilms, Gonçalves et al. [112] concluded that generally carboxymethylchitosan is more effective for preventing the formation of Gram-negative biofilm than Gram-positive. The cell wall of Gram-positive bacteria is characterized by the presence of an external layer of peptidoglycans and an internal layer (plasmic membrane) phospholipids-rich. On the opposite hand, Gram-negative bacteria have a cell wall constituted of phospholipids-rich layers intercalated with a thin peptidoglycan layer. These differences are used for the Gram differentiation, but they also reflect substantial differences in their interactions with antibacterial agents. In general, NPs are more active against Gram-negative bacteria than for Gram-positive ones. Indeed, the inhibitory effects of NPs is enhanced by the attraction between the anionic compounds (lipopolysaccharides, LPS) of bacterial cell wall and the positive charge of NPs [113,114,115].

The enhanced antibacterial activity of magnetite/chitosan could be attributed to the interaction between the cationic amino groups on chitosan with anionic components such as N-acetylmuramic acid, neuraminic acid, and sialic acid usually found in microbial cell wall. Several studies showed that the microbial growth is hampered through (a) enzyme inhibitions, (b) reduction in selective permeability, and (c) chelation of different transitional metal ions [62,116].

In the case of magnetic biochar loaded with quaternary phosphonium salt, Fu et al. [117] reported several mechanisms associated with the release of the phosphonium salt causes the disruption of the cell membrane and the increased permeability of compounds including magnetic nanoparticles (which increase the biocide effect). The oxidative damage inhibited the essential cell metabolism. In addition, the high antibacterial activity of PGG@MC could be related to the high stability of magnetite/chitosan in an aqueous solution, due to the protection effect of chitosan on NPs [70]. Several previous studies correlate the antibacterial activity of some compounds with the oxidative stress caused by the formation of reactive oxygen species (ROS) [64,118,119]. Several studies have shown that the phosphorylation of guar gum enhanced the antioxidant activity of the biopolymer [120,121]. The antibacterial effect of the composite results from these combined and crossed effects of phosphorylated guar gum and magnetic chitosan compartments.

Though the current preliminary investigation only focuses on bacteria, and limit the study of a unique concentration (while it would be necessary evaluating the limit concentration for antibacterial activity), this study shows that these materials have promising activity against bacterial contaminations. Many other investigations would be necessary to evaluate the innocuous nature of the composite before extending the application of these materials to biomedical and biological applications.

## 4. Conclusions

The sorption properties of the composite associating phosphorylated guar gum and chitosan (or magnetite NPs/chitosan) allows designing an efficient sorbent for uranyl recovery from low-concentration solutions at mild acidic conditions (i.e., pH~4). The initial slope of the sorption isotherm (modelled by the Langmuir equation) confirms the strong affinity of the material (especially non-magnetic composite) and the sorption capacity at saturation of the monolayer is consistent with the values reported, at this pH, for most of the efficient U(VI) sorbents. The presence of magnetite NPs slightly increases the speed of mass transfer: uptake kinetics is controlled by the pseudo-first order rate equation; although the resistance to intraparticle diffusion plays a significant role in the control of uptake kinetics. The selectivity tests performed on multi-component solutions (at equimolar concentration) showed the strong preference of the sorbents for U(VI) and Nd(III) against alkali-earth metals ions (Ca(II) and Mg(II)) and metalloid (Si(IV)). This selectivity is especially significant at pH close to the optimum pH value (i.e., pH_0_: 4): these elements are commonly found in the leachates of Egyptian ores from Sinai. These results illustrate the possibility to use these materials for the selective recovery of strategic metals from complex effluents (and from contaminated solutions and groundwater). The desorption is readily operated using 0.2 M HCl solutions: U(VI) elution is fast (equilibrium time around 20 min of contact against 60 min for the sorption step). In addition, the sorbent can be re-used for a minimum of five cycles with a loss in sorption performance lower than 6%. 

The application of the sorbents (especially PGG@C) for the recovery of U(VI) from washing pregnant solutions (generated by the water washing of Cl-rich uraniferous ores) allows the preferential recovery of U(VI) from other base and alkali-earth metals (and silicate ions). This high efficiency of PGG@C allows decreasing the environmental impact of this required washing pre-treatment of Egyptian ores (prior to acidic leaching and U(VI) recovery using quaternary ammonium salt resins).

A preliminary study (based on the determination of zones of inhibition, ZOI) showed that in addition to these outstanding sorption properties, the materials have promising antibacterial properties (which deserve complementary investigation for fixing the minimum inhibition concentration, etc.). Substantial differences are observed between PGG@C (efficient only against Gram-positive bacteria) and PGG@MC (with larger active spectrum). Although PGG@MC has little stronger antibacterial activity (ZOI was about 27% larger) against Gram–positive bacteria, the inhibitory effect is significant also against Gram-negative bacteria. 

## Figures and Tables

**Table 1:** Parameters of the PFORE for the modeling of uptake kinetics for the different sorbents.

Sorbent	Agitation Mode	q_eq,exp._ (mmol U g^−1^)	q_eq,1_ (mmol U g^−1^)	k_1_ × 10^2^ (min^−1^)	R^2^	AIC
Chit	MA	0.094	0.104	4.94	0.991	−141
GG	MA	0.070	0.075	5.19	0.971	−134
PGG@C #1	MA	0.488	0.512	7.83	0.995	−107
PGG@C #2	MA	0.481	0.510	7.54	0.990	−100
MChit	MA	0.065	0.067	10.4	0.985	−141
MGG	MA	0.062	0.064	9.34	0.989	−149
PGG@MC #1	MA	0.433	0.446	11.3	0.990	−103
PGG@MC #2	MA	0.438	0.454	10.5	0.986	−99
PGG@C	UT	0.501	0.501	25.6	0.998	−123
PGG@MC	UT	0.397	0.398	28.7	0.989	−106

MA: Mechanical Agitation; UT, Ultrasonic Treatment.

**Table 2:** Parameters of the Langmuir equation for the modeling of sorption isotherms for the different sorbents.

Sorbent	q_m,exp._ (mmol U g^−1^)	q_m,L._ (mmol U g^−1^)	b_L_ (L mmol^−1^)	q_m,L_ × b_L_ (L g^−1^)	R^2^	AIC
Chit	0.288	0.482	0.888	0.428	0.988	−92
GG	0.256	0.472	0.694	0.328	0.970	−85
PGG@C #1	1.29	1.33	46.6	62.1	0.969	−47
PGG@C #2	1.27	1.30	47.6	62.0	0.966	−47
MChit	0.239	0.494	0.541	0.267	0.979	−90
MGG	0.203	0.457	0.467	0.214	0.964	−88
PGG@MC #1	1.16	1.22	23.6	28.7	0.984	−57
PGG@MC #2	1.15	1.22	20.3	24.9	0.979	−55

**Table 3:** Sorbent recycling-Sorption and desorption efficiencies for 5 successive cycles

		Sorption		Desorption	
Sorbent	Cycle	Sorption Efficiency (%)	Std. Dev.	Desorption Efficiency (%)	Std. Dev.
PGG@C	1	97.0	0.1	100.0	0.0
	2	95.9	0.1	99.9	0.0
	3	94.1	0.2	100.0	0.1
	4	93.1	0.2	99.5	0.5
	5	92.2 [−5%]	0.1	98.9	0.3
PGG@MC	1	89.9	0.6	99.6	0.0
	2	89.1	0.4	100.0	0.0
	3	87.5	1.0	100.3	0.4
	4	85.5	0.6	100.1	0.0
	5	84.6 [−6%]	1.1	99.7	0.3

Sorption: C_0_: 0.42 mmol U L^−1^; pH_0_: 4; SD: 833 mg L^−1^; time: 24 h; T: 21 ± 1 °C. Desorption: 0.2 M HCl eluent; SD: 2.5 g L^−1^; time: 2 h; T: 21 ± 1 °C.

## Data Availability

Data are available from authors.

## Supplementary Materials

The following are available online. Table S1a: Reminder on equations used for modeling uptake kinetics, Table S1b: Reminder on equations used for modeling sorption isotherms, Table S2: Parameters of the PSORE for the modeling of uptake kinetics for the different sorbents, Table S3: Parameters of the RIDE (Effective Diffusivity, D_e_) for the modeling of uptake kinetics for the different sorbents, Table S4: Parameters of the Freundlich equation for the modeling of sorption isotherms for the different sorbents, Table S5: Parameters of the Sips equation for the modeling of sorption isotherms for the different sorbents, Table S6: U(VI) sorption properties of PGG@C and PGG@MC sorbents and alternative sorbents, Table S7: Composition of selected ore, Table S8: Composition of WPS, and sorption efficiency (%) at different pH values (using PGG@C and PGG@MC), Table S9: Ionic properties of selected elements (hydrated), Figure S1: SEM images of PGG@C and PGG@MC, Figure S2: Structural characterization of PGG@MC (magnetic behavior), and XRD diffraction patterns, Figure S3: Thermogravimetric analysis (TGA and DrTG) of PGG, PGG@C and PGG@MC, Figure S4: FTIR spectra of different functionalized materials and composites, Figure S5: FTIR spectra of PGG@C (a) and PGG@MC (b), after U(VI) sorption and after 5 cycles of recycling, Figure S6: Determination of pH_PZC_ of PGG@C and PGG@MC sorbents, Figure S7: U(VI) speciation–Effect of pH, Figure S8: pH variation during U(VI) sorption using PGG@C and PGG@MC (and intermediary products: Chit, MChit, GG, and MGG), Figure S9: Plot of log_10_ D (L g^−1^) vs. pH_eq_ for U(VI) sorption using PGG@C and PGG@MC. Figure S10: U(VI) uptake kinetics using Chit, GG, and PGG@C sorbents: non-magnetic (a) and magnetic (b) sorbents–Modeling with the PSORE, Figure S11: U(VI) uptake kinetics using Chit, GG, and PGG@C sorbents: non-magnetic (a) and magnetic (b) sorbents–Modeling with the PSORE, Figure S12: Effect of ultrasonic treatment (UT) on U(VI) uptake kinetics using PGG@C (a) and PGG@MC (b) sorbents–Modeling with the PSORE, Figure S13: Effect of ultrasonic treatment (UT) on U(VI) uptake kinetics using PGG@C (a) and PGG@MC (b) sorbents–Modeling with the RIDE, Figure S14: Effect of pH on the selectivity coefficients SC_(Nd/Me)_ and SC_(U/Me)_ for PGG@C and PGG@MC, Figure S15: Comparison of U(VI) desorption kinetics for selected sorbents, Figure S16: Geological and location map of mining area, Figure S17: Water washing of ore–Evolution of the concentrations of Cl^−^ (g L^−1^) and U(VI) (mg L^−1^) at the outlet of the column, Figure S18: Effect of pH_eq_ on sorption efficiency (%) for target elements from WPS using PGG@C and PGG@MC, Figure S19: Semi-quantitative EDX analyses of PCC@C and PGG@MC after contact with WPS.

## References

[1] Wu S., Zhao L., Wang L., Huang X., Zhang Y., Feng Z., Cui D. (2019). Simultaneous recovery of rare earth elements and phosphorus from phosphate rock by phosphoric acid leaching and selective precipitation: Towards green process. J. Rare Earths.

[2] Pavon S., Fortuny A., Coll M.T., Sastre A.M. (2018). Rare earths separation from fluorescent lamp wastes using ionic liquids as extractant agents. Waste Manag..

[3] Ni’am A.C., Wang Y.-F., Chen S.-W., Chang G.-M., You S.-J. (2020). Simultaneous recovery of rare earth elements from waste permanent magnets (WPMs) leach liquor by solvent extraction and hollow fiber supported liquid membrane. Chem. Eng. Process. Process Intensif..

[4] Wu S., Wang L., Zhang P., El-Shall H., Moudgil B., Huang X., Zhao L., Zhang L., Feng Z. (2018). Simultaneous recovery of rare earths and uranium from wet process phosphoric acid using solvent extraction with D2EHPA. Hydrometallurgy.

[5] Torkabad M.G., Keshtkar A.R., Safdari S.J. (2017). Comparison of polyethersulfone and polyamide nanofiltration membranes for uranium removal from aqueous solution. Prog. Nucl. Energy.

[6] Pavon S., Fortuny A., Call M.T., Sastre A.M. (2019). Improved rare earth elements recovery from fluorescent lamp wastes applying supported liquid membranes to the leaching solutions. Sep. Purif. Technol..

[7] Erkaya I.A., Arica M.Y., Akbulut A., Bayramoglu G. (2014). Biosorption of uranium(VI) by free and entrapped *Chlamydomonas reinhardtii*: Kinetic, equilibrium and thermodynamic studies. J. Radioanal. Nucl. Chem..

[8] Lapo B., Bou J.J., Hoyo J., Carrillo M., Pena K., Tzanov T., Sastre A.M. (2020). A potential lignocellulosic biomass based on banana waste for critical rare earths recovery from aqueous solutions. Environ. Pollut..

[9] Ferreira R.V.d.P., de Araujo L.G., Canevesi R.L.S., da Silva E.A., Ferreira E.G.A., Palmieri M.C., Marumo J.T. (2020). The use of rice and coffee husks for biosorption of U (total), Am-241, and(137)Cs in radioactive liquid organic waste. Environ. Sci. Pollut. Res..

[10] Javadian H., Ruiz M., Saleh T.A., Sastre A.M. (2020). Ca-alginate/carboxymethyl chitosan/Ni_0.2_Zn_0.2_F_2.6_O_4_ magnetic bionanocomposite: Synthesis, characterization and application for single adsorption of Nd^+3^, Tb^+3^, and Dy^+^3 rare earth elements from aqueous media. J. Mol. Liq..

[11] Javadian H., Ruiz M., Sastre A.M. (2020). Response surface methodology based on central composite design for simultaneous adsorption of rare earth elements using nanoporous calcium alginate/carboxymethyl chitosan microbiocomposite powder containing Ni_0.2_Zn_0.2_Fe_2.6_O_4_ magnetic nanoparticles: Batch and column studies. Int. J. Biol. Macromol..

[12] Ma F.Q., Dong B.R., Gui Y.Y., Cao M., Han L., Jiao C.S., Lv H.T., Hou J.J., Xue Y. (2018). Adsorption of low-concentration uranyl ion by amidoxime polyacrylonitrile fibers. Ind. Eng. Chem. Res..

[13] Wiechert A.I., Liao W.-P., Hong E., Halbert C.E., Yiacoumi S., Saito T., Tsouris C. (2018). Influence of hydrophilic groups and metal-ion adsorption on polymer-chain conformation of amidoxime-based uranium adsorbents. J. Colloid Interface Sci..

[14] Wongjaikham W., Wongsawaeng D., Hosemann P. (2019). Synthesis of amidoxime polymer gel to extract uranium compound from seawater by UV radiation curing. J. Nucl. Sci. Technol..

[15] Wu H.Y., Chi F.T., Zhang S., Wen J., Xiong J., Hu S. (2019). Control of pore chemistry in metal-organic frameworks for selective uranium extraction from seawater. Microporous Mesoporous Mater..

[16] Hamza M.F., Gamal A., Hussein G., Nagar M.S., Abdel-Rahman A.A.H., Wei Y., Guibal E. (2019). Uranium(VI) and zirconium(IV) sorption on magnetic chitosan derivatives-effect of different functional groups on separation properties. J. Chem. Technol. Biotechnol..

[17] He D.X., Tan N., Luo X.M., Yang X.C., Ji K., Han J.W., Chen C., Liu Y.Q. (2020). Preparation, uranium (VI) absorption and reuseability of marine fungus mycelium modified by the bis-amidoxime-based groups. Radiochim. Acta.

[18] Dousti Z., Dolatyari L., Yaftian M.R., Rostamnia S. (2019). Adsorption of Eu(III), Th(IV), and U(VI) by mesoporous solid materials bearing sulfonic acid and sulfamic acid functionalities. Sep. Sci. Technol..

[19] Taha M.H. (2020). Solid-liquid extraction of uranium from industrial phosphoric acid using macroporous cation exchange resins: MTC1600H, MTS9500, and MTS9570. Sep. Sci. Technol..

[20] Ahmad M., Yang K., Li L., Fan Y., Shah T., Zhang Q., Zhang B. (2020). Modified tubular carbon nanofibers for adsorption of uranium(VI) from water. ACS Appl. Nano Mater..

[21] Riegel M., Schlitt V. (2017). Sorption dynamics of uranium onto anion exchangers. Water.

[22] Masoud A.M. (2020). Sorption behavior of uranium from sulfate media using purolite A400 as a strong base anion exchange resin. Int. J. Environ. Anal. Chem..

[23] Hamza M.F., Mubark A.E., Wei Y., Vincent T., Guibal E. (2020). Quaternization of composite algal/PEI beads for enhanced uranium sorption-application to ore acidic leachate. Gels.

[24] Ang K.L., Li D., Nikoloski A.N. (2018). The effectiveness of ion exchange resins in separating uranium and thorium from rare earth elements in acidic aqueous sulfate media. Part 2. Chelating resins. Miner. Eng..

[25] Guo X., Feng Y., Ma L., Gao D., Jing J., Yu J., Sun H., Gong H., Zhang Y. (2017). Phosphoryl functionalized mesoporous silica for uranium adsorption. Appl. Surf. Sci.

[26] Sarafraz H., Minuchehr A., Alahyarizadeh G., Rahimi Z. (2017). Synthesis of enhanced phosphonic functional groups mesoporous silica for uranium selective adsorption from aqueous solutions. Sci. Rep..

[27] Xu M.Y., Han X.L., Wang T., Li S.H., Hua D.B. (2018). Conjugated microporous polymers bearing phosphonate ligands as an efficient sorbent for potential uranium extraction from high- level liquid wastes. J. Mater. Chem. A.

[28] Duval C.E., Hardy W.A., Pellizzeri S., DeVol T.A., Husson S.M. (2019). Phosphonic acid and alkyl phosphate-derivatized resins for the simultaneous concentration and detection of uranium in environmental waters. React. Funct. Polym..

[29] Yang P.P., Liu Q., Liu J.Y., Chen R.R., Li R.M., Bai X.F., Wang J. (2019). Highly efficient immobilization of uranium(VI) from aqueous solution by phosphonate-functionalized dendritic fibrous nanosilica (DFNS). J. Hazard. Mater..

[30] Yuan D.Z., Zhang S.A., Tan J.L., Dai Y.H., Wang Y., He Y., Liu Y., Zhao X.H., Zhang M.M., Zhang Q.H. (2020). Highly efficacious entrapment of Th (IV) and U (VI) from rare earth elements in concentrated nitric acid solution using a phosphonic acid functionalized porous organic polymer adsorbent. Sep. Purif. Technol..

[31] Horwitz E.P., Chiarizia R., Diamond H., Gatrone R.C., Alexandratos S.D., Trochimczuk A.Q., Crick D.W. (1993). Uptake of metal-ions by a new chelating ion-exchange resin. 1. Acid dependencies of actinide ions. Solvent Extr. Ion Exch..

[32] Sabharwal K.N., Nandy K.K., Srinivasan T.G., Rao P.R.V. (1996). Recovery of uranium from acid media by macroporous bifunctional phosphinic acid resin. Solvent Extr. Ion Exch..

[33] Sabharwal K.N., Rao P.R.V., Srinivasan M. (1995). Extraction of uranium by macroporous bifunctional phosphinic acid resin. Solvent Extr. Ion Exch..

[34] Bhanushali R.D., Pius I.C., Chetty K.V., Vaidya V.N., Venugopal V., Rao P.R.V. (2005). Sorption of Pu(IV) on macroporous bifunctional phosphinic acid resin from uranium analytical waste solution. J. Radioanal. Nucl. Chem..

[35] Venkatesan K.A., Patre D.K., Sabharwal K.N., Srinivasan T.G., Rao P.R.V. (2000). Kinetics of uranium extraction by macroporous bifunctional phosphinic acid resin. Solvent Extr. Ion Exch..

[36] Piechowicz M., Abney C.W., Zhou X., Thacker N.C., Li Z., Lin W. (2016). Design, synthesis, and characterization of a bifunctional chelator with ultrahigh capacity for uranium uptake from seawater simulant. Ind. Eng. Chem. Res..

[37] Wei Y.Q., Qian J., Huang L., Hua D.B. (2015). Bifunctional polymeric microspheres for efficient uranium sorption from aqueous solution: Synergistic interaction of positive charge and amidoxime group. RSC Adv..

[38] Alexandratos S.D., Zhu X. (2016). Polymer-supported aminomethylphosphinate as a ligand with a high affinity for U(VI) from phosphoric acid solutions: Combining variables to optimize ligand-ion communication. Solvent Extr. Ion Exch..

[39] Rashad M.M., El-Sayed I.E., Galhoum A.A., Abdeen M.M., Mira H.I., Elshehy E.A., Zhang S., Lu X., Xin J., Guibal E. (2020). Synthesis of α-aminophosphonate based sorbents–Influence of inserted groups (carboxylic vs. amine) on uranyl sorption. Chem. Eng. J..

[40] Cheira M.F. (2020). Synthesis of aminophosphonate-functionalised ZnO/polystyrene-butadiene nanocomposite and its characteristics for uranium adsorption from phosphoric acid. Int. J. Environ. Anal. Chem..

[41] Imam E.A., El-Tantawy El-Sayed I., Mahfouz M.G., Tolba A.A., Akashi T., Galhoum A.A., Guibal E. (2018). Synthesis of α-aminophosphonate functionalized chitosan sorbents: Effect of methyl vs phenyl group on uranium sorption. Chem. Eng. J..

[42] Vaaramaa K., Pulli S., Lehto J. (2000). Effects of pH and uranium concentration on the removal of uranium from drinking water by ion exchange. Radiochim. Acta.

[43] Pawar S.N., Edgar K.J. (2012). Alginate derivatization: A review of chemistry, properties and applications. Biomaterials.

[44] Guibal E. (2004). Interactions of metal ions with chitosan-based sorbents: A review. Sep. Purif. Technol..

[45] Zhu Y.H., Hu J., Wang J.L. (2012). Competitive adsorption of Pb(II), Cu(II) and Zn(II) onto xanthate-modified magnetic chitosan. J. Hazard. Mater..

[46] Yuvaraja G., Su M., Chen D.-Y., Pang Y., Kong L.-J., Subbaiah M.V., Wen J.-C., Reddy G.M. (2020). Impregnation of magnetic–*Momordica charantia* leaf powder into chitosan for the removal of U(VI) from aqueous and polluted wastewater. Int. J. Biol. Macromol..

[47] Wang Y., Li Y., Li L., Kong F., Lin S., Wang Z., Li W. (2020). Preparation of three-dimensional fiber-network chitosan films for the efficient treatment of uranium-contaminated effluents. Water Sci. Technol..

[48] Hamza M.F., Roux J.-C., Guibal E. (2018). Uranium and europium sorption on amidoxime-functionalized magnetic chitosan micro-particles. Chem. Eng. J..

[49] Jakobik-Kolon A., Bok-Badura J., Milewski A., Karon K. (2019). Long term and large-scale continuous studies on zinc(II) sorption and desorption on hybrid pectin-guar gum biosorbent. Polymers.

[50] Singha N.R., Mahapatra M., Karmakar M., Dutta A., Mondal H., Chattopadhyay P.K. (2017). Synthesis of guar gum-g-(acrylic acid-co-acrylamide-co-3-acrylamido propanoic acid) IPN via in situ attachment of acrylamido propanoic acid for analyzing superadsorption mechanism of Pb(II)/Cd(II)/Cu(II)/MB/MV. Polym. Chem..

[51] Patra A.S., Ghorai S., Sarkar D., Das R., Sarkar S., Pal S. (2017). Anionically functionalized guar gum embedded with silica nanoparticles: An efficient nanocomposite adsorbent for rapid adsorptive removal of toxic cationic dyes and metal ions. Bioresour. Technol..

[52] Dai L., Zhang L., Wang B., Yang B., Khan I., Khan A., Ni Y. (2017). Multifunctional self-assembling hydrogel from guar gum. Chem. Eng. J..

[53] Hiremath J.N., Vishalakshi B. (2016). Effective removal of divalent metal ions: Synthesis and characterization of pH-sensitive guar gum based hydrogels. Desalin. Water Treat..

[54] Thakur S., Kumari S., Dogra P., Chauhan G.S. (2014). A new guar gum-based adsorbent for the removal of Hg(II) from its aqueous solutions. Carbohydr. Polym..

[55] Fresco-Cala B., Batista A.D., Cardenas S. (2020). Molecularly imprinted polymer micro- and nano-particles: A review. Molecules.

[56] Habila M.A., Alothman Z.A., El-Toni A.M., Labis J.P., Khan A., Al-Marghany A., Elafifi H.E. (2017). One-step carbon coating and polyacrylamide functionalization of Fe_3_O_4_ nanoparticles for enhancing magnetic adsorptive-remediation of heavy metals. Molecules.

[57] Manousi N., Rosenberg E., Deliyanni E., Zachariadis G.A., Samanidou V. (2020). Magnetic solid-phase extraction of organic compounds based on graphene oxide nanocomposites. Molecules.

[58] Xu M., Han X., Hua D. (2017). Polyoxime-functionalized magnetic nanoparticles for uranium adsorption with high selectivity over vanadium. J. Mater. Chem. A.

[59] Hamza M.F., Wei Y., Mira H.I., Abdel-Rahman A.A.H., Guibal E. (2019). Synthesis and adsorption characteristics of grafted hydrazinyl amine magnetite-chitosan for Ni(II) and Pb(II) recovery. Chem. Eng. J..

[60] Liakos I., Grumezescu A.M., Holban A.M. (2014). Magnetite nanostructures as novel strategies for anti-infectious therapy. Molecules.

[61] Haldorai Y., Kharismadewi D., Tuma D., Shim J.-J. (2015). Properties of chitosan/magnetite nanoparticles composites for efficient dye adsorption and antibacterial agent. Korean J. Chem. Eng..

[62] Salem S.S., Fouda A. (2021). Green synthesis of metallic nanoparticles and their prospective biotechnological applications: An overview. Biol. Trace Elem. Res..

[63] Yeswanth S., Sekhar K.C., Chaudhary A., Sarma P. (2018). Anti-microbial and Anti-biofilm activity of a novel Dibenzyl (benzo d thiazol-2-yl (hydroxy) methyl) phosphonate by inducing protease expression in *Staphylococcus aureus*. Med. Chem. Res..

[64] Fouda A., Hassan S.E.-D., Abdo A.M., El-Gamal M.S. (2020). Antimicrobial, antioxidant and larvicidal activities of spherical silver nanoparticles synthesized by endophytic *Streptomyces* spp.. Biol. Trace Elem. Res..

[65] Massart R. (1981). Preparation of aqueous magnetic liquids in alkaline and acidic media. IEEE Trans. Magn..

[66] Marczenko Z., Balcerzak M., Marczenko Z., Balcerzak M. (2000). Chapter 54-Uranium. Analytical Spectroscopy Library.

[67] Marczenko Z., Balcerzak M., Marczenko Z., Balcerzak M. (2000). Chapter 39-Rare-earth Elements. Analytical Spectroscopy Library.

[68] Davies W., Gray W. (1964). A rapid and specific titrimetric method for the precise determination of uranium using iron(II) sulphate as reductant. Talanta.

[69] Mathew K.J., Buerger S., Vogt S., Mason P., Morales-Arteaga M.E., Narayanan U.I. (2009). Uranium assay determination using Davies and Gray titration: An overview and implementation of GUM for uncertainty evaluation. J. Radioanal. Nucl. Chem..

[70] Nehra P., Chauhan R.P., Garg N., Verma K. (2018). Antibacterial and antifungal activity of chitosan coated iron oxide nanoparticles. Br. J. Biomed. Sci..

[71] Salem N., Ahmad A., Awwad A. (2013). New route for synthesis magnetite nanoparticles from ferrous ions and Pistachio leaf extract. J. Nanosci. Nanotechnol..

[72] Stoia M., Istratie R., Păcurariu C. (2016). Investigation of magnetite nanoparticles stability in air by thermal analysis and FTIR spectroscopy. J. Therm. Anal. Calorim..

[73] Wang S., Yu D. (2010). Adsorption of Cd(II), Pb(II), and Ag(I) in aqueous solution on hollow chitosan microspheres. J. Appl. Polym. Sci..

[74] Duarte M.L., Ferreira M.C., Marvao M.R., Rocha J. (2002). An optimised method to determine the degree of acetylation of chitin and chitosan by FTIR spectroscopy. Int. J. Biol. Macromol..

[75] Mudgil D., Barak S., Khatkar B.S. (2012). X-ray diffraction, IR spectroscopy and thermal characterization of partially hydrolyzed guar gum. Int. J. Biol. Macromol..

[76] Coleman R.J., Lawrie G., Lambert L.K., Whittaker M., Jack K.S., Grøndahl L. (2011). Phosphorylation of alginate: Synthesis, characterization, and evaluation of in vitro mineralization capacity. Biomacromolecules.

[77] Petcharoen K., Sirivat A. (2012). Synthesis and characterization of magnetite nanoparticles via the chemical co-precipitation method. Mater. Sci. Eng. B.

[78] Sorlier P., Denuzière A., Viton C., Domard A. (2001). Relation between the degree of acetylation and the electrostatic properties of chitin and chitosan. Biomacromolecules.

[79] Pal A., Das T., Sengupta S., Sardar S., Mondal S., Bandyopadhyay A. (2020). An elastic semi IPN polymer hybrid for enhanced adsorption of heavy metals. Carbohydr. Polym..

[80] Zhang W., Bu A., Ji Q.Y., Min L.F., Zhao S., Wang Y.X., Chen J. (2019). pK_a_-directed incorporation of phosphonates into MOF-808 via ligand exchange: Stability and adsorption properties for uranium. ACS Appl. Mater. Interfaces.

[81] Singha N.R., Dutta A., Mahapatra M., Karmakar M., Mondal H., Chattopadhyay P.K., Maiti D.K. (2018). Guar gum-grafted terpolymer hydrogels for ligand-selective individual and synergistic adsorption: Effect of comonomer composition. ACS Omega.

[82] Yean S., Cong L., Yavuz C.T., Mayo J.T., Yu W.W., Kan A.T., Colvin V.L., Tomson M.B. (2005). Effect of magnetite particle size on adsorption and desorption of arsenite and arsenate. J. Mater. Res..

[83] Thomas G., Demoisson F., Boudon J., Millot N. (2016). Efficient functionalization of magnetite nanoparticles with phosphonate using a one-step continuous hydrothermal process. Dalton Trans..

[84] Abu-Dalo M., Al-Rawashdeh N., Al-Mheidat I., Nassory N. (2015). Construction of uranyl selective electrode based on complex of uranyl ion with new ligand carboxybenzotriazole in PVC matrix membrane. IOP Conference Series Materials Science and Engineering.

[85] Krishna G.M., Suneesh A.S., Kumaresan R., Venkatesan K.A., Antony M.P. (2017). Electrochemical behavior of U(VI) in imidazolium ionic liquid medium containing tri-n-butyl phosphate and chloride ion and spectroscopic characterization of uranyl species. ChemistrySelect.

[86] Guibal E., Roulph C., Lecloirec P. (1992). Uranium biosorption by a filamentous fungus *Mucor miehei*: pH effect on mechanisms and performances of uptake. Water Res..

[87] Crank J. (1975). The Mathematics of Diffusion.

[88] Ho Y.S., McKay G. (1999). Pseudo-second order model for sorption processes. Process Biochem..

[89] Simonin J.-P. (2016). On the comparison of pseudo-first order and pseudo-second order rate laws in the modeling of adsorption kinetics. Chem. Eng. J..

[90] Hubbe M.A., Azizian S., Douven S. (2019). Implications of apparent pseudo-second-order adsorption kinetics onto cellulosic materials: A review. BioResources.

[91] Awakura Y., Sato K., Majima H., Hirono S. (1987). The measurement of the diffusion coefficient of U(VI) in aqueous uranyl sulfate solutions. Metall. Trans. B.

[92] Wen Z., Huang K., Niu Y., Yao Y., Wang S., Cao Z., Zhong H. (2020). Kinetic study of ultrasonic-assisted uranium adsorption by anion exchange resin. Colloids Surf. A.

[93] Elwakeel K.Z., Hamza M.F., Guibal E. (2021). Effect of agitation mode (mechanical, ultrasound and microwave) on uranium sorption using amine- and dithizone-functionalized magnetic chitosan hybrid materials. Chem. Eng. J..

[94] Amesh P., Venkatesan K.A., Suneesh A.S., Samanta N. (2020). Diethylenetriamine tethered mesoporous silica for the sequestration of uranium from aqueous solution and seawater. J. Environ. Chem. Eng..

[95] Huo Z., Zhao S., Yi J., Zhang H., Li J. (2020). Biomass-based cellulose functionalized by phosphonic acid with high selectivity and capacity for capturing U(VI) in aqueous solution. Appl. Sci..

[96] Venkatesan K.A., Shyamala K.V., Antony M.P., Srinivasan T.G., Rao P.R.V. (2008). Batch and dynamic extraction of uranium(VI) from nitric acid medium by commercial phosphinic acid resin, Tulsion CH-96. J. Radioanal. Nucl. Chem..

[97] Zhang S., Yuan D., Zhang Q., Wang Y., Liu Y., Zhao J., Chen B. (2020). Highly efficient removal of uranium from highly acidic media achieved using a phosphine oxide and amino functionalized superparamagnetic composite polymer adsorbent. J. Mater. Chem. A.

[98] Persson I. (2010). Hydrated metal ions in aqueous solution: How regular are their structures?. Pure Appl. Chem..

[99] Marcus Y. (1997). Ion Properties.

[100] Li K., Li M., Xue D. (2012). Solution-phase electronegativity scale: Insight into the chemical behaviors of metal ions in solution. J. Phys. Chem. A.

[101] Merdivan M., Buchmeiser M.R., Bonn G. (1999). Phosphonate-based resins for the selective enrichment of uranium(VI). Anal. Chim. Acta.

[102] Xu H., Xu D.C., Wang Y. (2017). Natural indices for the chemical hardness/softness of metal cations and ligands. ACS Omeg.

[103] Yang Y.J., Alexandratos S.D. (2009). Affinity of polymer-supported reagents for lanthanides as a function of donor atom polarizability. Ind. Eng. Chem. Res..

[104] Sirry S.M., Aldakhil F., Alharbi O.M.L., Ali I. (2019). Chemically treated date stones for uranium (VI) uptake and extraction in aqueous solutions. J. Mol. Liq..

[105] Oyola Y., Vukovic S., Dai S. (2016). Elution by Le Chatelier’s principle for maximum recyclability of adsorbents: Applied to polyacrylamidoxime adsorbents for extraction of uranium from seawater. Dalton Trans..

[106] Kabay N., Demircioglu M., Yayli S., Gunay E., Yuksel M., Saglam M., Streat M. (1998). Recovery of uranium from phosphoric acid solutions using chelating ion-exchange resins. Ind. Eng. Chem. Res..

[107] Stopa L.C.B., Yamaura M. (2010). Uranium removal by chitosan impregnated with magnetite nanoparticles: Adsorption and desorption. Int. J. Nucl. Energy Sci. Technol..

[108] Pan H.-B., Wai C.M., Kuo L.-J., Gill G., Tian G., Rao L., Das S., Mayes R.T., Janke C.J. (2017). Bicarbonate elution of uranium from amidoxime-based polymer adsorbents for sequestering uranium from seawater. Chemistryselect.

[109] Hamza M.F. (2018). Uranium recovery from concentrated chloride solution produced from direct acid leaching of calcareous shale, Allouga ore materials, southwestern Sinai, Egypt. J. Radioanal. Nucl. Chem..

[110] Merritt R.C. (1971). The Extractive Metallurgy of Uranium.

[111] Pearson R.G. (1966). Acids and bases. Science.

[112] Goncalves R.C., da Silva D.P., Signini R., Naves P.L.F. (2017). Inhibition of bacterial biofilms by carboxymethyl chitosan combined with silver, zinc and copper salts. Int. J. Biol. Macromol..

[113] Abbaszadegan A., Ghahramani Y., Gholami A., Hemmateenejad B., Dorostkar S., Nabavizadeh M., Sharghi H. (2015). The effect of charge at the surface of silver nanoparticles on antimicrobial activity against Gram-positive and Gram-negative bacteria: A peliminary study. J. Nanomater..

[114] Roy A., Bulut O., Some S., Mandal A.K., Yilmaz M.D. (2019). Green synthesis of silver nanoparticles: Biomolecule-nanoparticle organizations targeting antimicrobial activity. RSC Adv..

[115] Hassan S.E.-D., Fouda A., Radwan A.A., Salem S.S., Barghoth M.G., Awad M.A., Abdo A.M., El-Gamal M.S. (2019). Endophytic actinomycetes *Streptomyces* spp mediated biosynthesis of copper oxide nanoparticles as a promising tool for biotechnological applications. J. Biol. Inorg. Chem..

[116] Shrifian-Esfahni A., Salehi M.T., Nasr-Esfahni M., Ekramian E. (2015). Chitosan-modified superparamgnetic iron oxide nanoparticles: Design, fabrication, characterization and antibacterial activity. Chemik.

[117] Fu Y., Wang F., Sheng H., Xu M., Liang Y., Bian Y., Hashsham S.A., Jiang X., Tiedje J.M. (2020). Enhanced antibacterial activity of magnetic biochar conjugated quaternary phosphonium salt. Carbon.

[118] Behera S., Patra J.K., Pramanik K., Panda N., Thatoi H. (2012). Characterization and evaluation of antibacterial activities of chemically synthesized iron oxide nanoparticles. World J. Nano Sci. Eng..

[119] El-Belely E.F., Farag M.M.S., Said H.A., Amin A.S., Azab E., Gobouri A.A., Fouda A. (2021). Green synthesis of zinc oxide nanoparticles (ZnO-NPs) using *Arthrospira platensis* (Class: *Cyanophyceae*) and evaluation of their biomedical activities. Nanomaterials.

[120] Niu S., Wang J., Zhao B., Zhao M., Nie M., Wang X., Yao J., Zhang J. (2013). Regioselective synthesis and antioxidant activities of phosphorylated guar gum. Int. J. Biol. Macromol..

[121] Wang J.L., Yang T., Tian J., Zeng T., Wang X.F., Yao J., Zhang J., Lei Z.Q. (2014). Synthesis and characterization of phosphorylated galactomannan: The effect of DS on solution conformation and antioxidant activities. Carbohydr. Polym..

