# Supplementary material for: Phosphorylation of Guar Gum/Magnetite/Chitosan Nanocomposites for Uranium (VI) Sorption and Antibacterial Applications
Source: Molecules. 2021 Mar 29;26(7):1920. doi: 10.3390/molecules26071920 (PMC8036802; doi:10.3390/molecules26071920)
Supplement: Supplementary file 1 [file molecules-26-01920-s001.pdf]

molecules

MDPI

Supplementary Information

Phosphorylation of guar gum/magnetite/chitosan nanocomposite for U(VI) sorption and antibacterial applications

Mohammed F. Hamza[1,2], Amr Fouda[3], Khalid Z. Elwakeel[4,5], Yuezhou Wei[1,6*], Eric Guibal[7*], Nora A. Hamad[8]

[1] Guangxi Key Laboratory of Processing for Non-ferrous Metals and Featured Materials, School of Resources, Environment and Materials, Guangxi University, Nanning 530004, PR China.; m_fouda21@hotmail.com (MFH) and yzwei@gxu.edu.cn (YW)

[2] Nuclear Materials Authority, POB 530, El-Maadi, Cairo, Egypt .; m_fouda21@hotmail.com (MFH)

[3] Botany and Microbiology Department, Faculty of Science, Al-Azhar University, Nasr City, Cairo 11884, Egypt; amr_fh83@azhar.edu.eg (AF)

[4] University of Jeddah, College of Science, Department of Chemistry, Jeddah, Saudi Arabia; kelwkeel@uj.edu.sa (KZE)

[5] Environmental Science Department, Faculty of Science, Port-Said University, Port-Said, Egypt; kelwkeel@uj.edu.sa (KZE)

[6] School of Nuclear Science and Engineering, Shanghai Jiao Tong University, Shanghai, China; yzwei@gxu.edu.cn (YW)

[7] Polymers Composites and Hybrids (PCH), IMT Mines Ales, Alès, France; eric.guibal@mines-ales.fr (EG)

[8] Faculty of Science, Menoufia University, Shebine El-Koam, Egypt; nhamad059@gmail.com.

* Correspondence: eric.guibal@mines-ales.fr (EG); yzwei@gxu.edu.cn (YW)

Table S1a. Reminder on equations used for modeling uptake kinetics [1, 2].

Model	Equation	Parameters	Ref.
PFORE	$q(t) = q_{eq,1}(1 - e^{-k_1 t})$	$q_{eq,1}$ (mmol g^{-1}): sorption capacity at equilibrium<br>k_1 (min^{-1}): apparent rate constant of PFORE	[1]
PSORE	$q(t) = \frac{q_{eq,2}^2 k_2 t}{1 + k_2 q_{eq,2} t}$	$q_{eq,2}$ (mmol g^{-1}): sorption capacity at equilibrium<br>k_2 (g $mmol^{-1}$ min^{-1}): apparent rate constant of PSORE	[1]
RIDE	$\frac{q(t)}{q_{eq}} = 1 - \sum_{n=1}^{\infty} \frac{6\alpha(\alpha+1)\exp\left(\frac{-D_e q_n^2}{r^2} t\right)}{9 + 9\alpha + q_n^2 \alpha^2}$<br>With q_n being the non-zero roots of<br>$\tan q_n = \frac{3\ q_n}{3 +\ \alpha q_n^2}$ and $\frac{m\ q}{V\ C_0} = \frac{1}{1\ +\ \alpha}$	D_e (m^2 min^{-1}) : Effective diffusivity coefficient	[2]

(m (g): mass of sorbent; V (L): volume of solution; C_0 (mmol L^{-1}): initial concentration of the solution).

Table S1b. Reminder on equations used for modeling sorption isotherms [2-4].

Model	Equation	Parameters	Ref.
Langmuir	$q_{eq} = \frac{q_{m,L} C_{eq}}{1 + b_L C_{eq}}$	$q_{m,L}$ (mmol g^{-1}): Sorption capacity at saturation of monolayer<br>b_L (L $mmol^{-1}$): Affinity coefficient	[3]
Freundlich	$q_{eq} = \quad k_F C_{eq}^{1/n_F}$	k_F and n_F: empirical parameters of Freundlich equation	[4]
Sips	$q_{eq} = \frac{q_{m,S} b_S C_{eq}^{1/n_S}}{1 + b_S C_{eq}^{1/n_S}}$	$q_{m,S}$, b_S and n_S: empirical parameters of Sips equation (based on Langmuir and Freundlich equations)	[2]

Akaike Information Criterion, AIC [5]:

$$AIC = N \ln\left(\frac{\sum_{i=0}^{N}\left(y_{i,exp.} - y_{i,model}\right)^2}{N}\right) + 2N_p + \frac{2N_p\left(N_p + 1\right)}{N - N_p - 1}$$

Where N is the number of experimental points, N_p the number of model parameters, $y_{i,exp.}$ and $y_{i,model}$ the experimental and calculated values of the tested variable.

Table S2. Parameters of the PSORE for the modeling of uptake kinetics for the different sorbents.

Sorbent	Agitation mode	$q_{eq,exp.}$ (mmol U g^{-1})	$q_{eq,2}$ (mmol U g^{-1})	$k_2 \times 10$ (min^{-1})	R^2	AIC
Chit	MA	0.094	0.412	2.92	0.985	-134
GG	MA	0.070	0.098	4.91	0.970	-134
PGG@C #1	MA	0.488	0.641	1.23	0.982	-92
PGG@C #2	MA	0.481	0.645	1.14	0.975	-89
MChit	MA	0.065	0.081	13.8	0.961	-131
MGG	MA	0.062	0.077	13.4	0.975	-139
PGG@MC #1	MA	0.433	0.529	2.45	0.968	-89
PGG@MC #2	MA	0.438	0.544	2.15	0.964	-88
PGG@C	UT	0.501	0.547	6.84	0.980	-94
PGG@MC	UT	0.397	0.432	9.85	0.959	-91

MA: Mechanical Agitation; UT, Ultrasonic Treatment.

Table S3. Parameters of the RIDE (Effective Diffusivity, D_e) for the modeling of uptake kinetics for the different sorbents.

**Sorbent**	**Agitation mode**	**$D_e \times 10^{12}$ (m^2 min^{-1})**	**R^2**	**AIC**
Chit	MA	11.7	0.970	-123
GG	MA	11.6	0.960	-133
PGG@C #1	MA	13.3	0.975	-86
PGG@C #2	MA	13.3	0.967	-83
MChit	MA	5.29	0.965	-133
MGG	MA	4.42	0.977	-140
PGG@MC #1	MA	4.82	0.970	-89
PGG@MC #2	MA	4.56	0.965	-87
PGG@C	UT	37.4	0.989	-103
PGG@MC	UT	11.4	0.973	-98

MA: Mechanical Agitation; UT, Ultrasonic Treatment.

Table S4. Parameters of the Freundlich equation for the modeling of sorption isotherms for the different sorbents.

Sorbent	k_F ($mmol^{1-1/n_F}$ L^{1/n_F} g^{-1})	n_F	R^2	AIC
Chit	0.215	1.640	0.981	-87
GG	0.185	1.572	0.969	-85
PGG@C #1	1.449	3.984	0.859	-31
PGG@C #2	1.419	4.106	0.869	-32
MChit	0.167	1.469	0.978	-90
MGG	0.140	1.419	0.963	-87
PGG@MC #1	1.256	3.593	0.891	-36
PGG@MC #2	1.255	3.530	0.890	-36

Table S5. Parameters of the Sips equation for the modeling of sorption isotherms for the different sorbents.

Sorbent	$q_{m,exp.}$ (mmol U g^{-1})	$q_{m,S.}$ (mmol U g^{-1})	b_S (L $mmol^{-1}$)	n_S	R^2	AIC
Chit	0.288	0.498	0.830	1.025	0.988	-88
GG	0.256	0.716	0.361	1.216	0.972	-82
PGG@C #1	1.29	1.28	158	0.795	0.972	-45
PGG@C #2	1.27	1.29	63.4	0.941	0.966	-43
MChit	0.239	0.709	0.318	1.157	0.979	-87
MGG	0.203	0.655	0.281	1.140	0.965	-84
PGG@MC #1	1.16	1.18	50.2	0.831	0.987	-55
PGG@MC #2	1.15	1.19	35.1	0.862	0.981	-51

Table S6. U(VI) sorption properties of PGG@C and PGG@MC sorbents and alternative sorbents (t_{eq}, min; q_m: mmol U g^{-1}; b_L: L $mmol^{-1}$).

Sorbent	pH	t_{eq}	$q_{m,exp}$	$q_{m,L.}$	b_L	Ref.
Tulsion CH-96 (in HNO_3 solution)	4 M	600	0.315	0.294	1.48	[6]
Magn. phosphine oxide/amino funct. composite	0.5	180	0.714	0.826	5.48	[7]
Tri-octylamine impregnated Siplite LX-16 resin	1.5	60	0.390	0.403	0.93	[8]
201 × 8 anion-exchange resin (on leachates)	1.57	180	-	0.282	5.71	[9]
Purolite A400 (strong base anion-exchanger)	1.9	480	-	0.494	61.9	[10]
Quinoline-silicate Lewatit resin	2.5	30	0.870	0.913	7.78	[11]
Duolite ES-467	3	90	-	0.326	10.9	[12]
Dowex 50WX8 / Alizarin Red-S	3	30	0.508	0.512	141	[13]
Amberlite IRA-402 (trimethyl ammonium)	3	120	-	0.894	11.9	[14]
Amberlite GG-400 anion exchange resin	3.5	360	-	0.240	57.2	[15]
Magnetic *Momordica charantia* leaf/chitosan	5	200	0.987	1.05	8.57	[16]
Cyanobacterial bloom (*Anabaena flos-aquae*)	5	60	0.825	0.799	28.6	[17]
Trioctyl amine impregnated MnO_2/zeolite	4	20	0.399	0.416	19.0	[18]
Aluminum sludge/PVA/sodium alginate	4	240	-	0.176	249	[19]
Hydroxypyridone-functionalized PE fabrics	4	1200	0.084	0.087	119	[20]
α-aminophosphonate resin	4	90	1.06	1.31	7.32	[21]
Magnetic poly(aminophosphonic) PGMA	4	240	1.12	1.20	16.2	[22]
Magnetic amidoxime-functionalized chitosan	4	90	1.50	1.57	53.9	[23]
Methyl-α-aminophosphonate funct. chitosan	4	120	1.12	1.35	6.64	[24]
Chit	*4*	*60*	*0.288*	*0.482*	*0.89*	*This work*
MChit	*4*	*60*	*0.239*	*0.494*	*0.54*	
GG	*4*	*60*	*0.256*	*0.472*	*0.69*	
MGG	*4*	*60*	*0.203*	*0.457*	*0.47*	
PGG@C	*4*	*60*	*1.28*	*1.32*	*47.1*	
PGG@MC	*4*	*60*	*1.15*	*1.22*	*22.0*	
Montmorillonite/Polyamide 6 composite	4.5	30	0.563	0.584	9.76	[25]
β-cyclodextrin functionalized silica gel	4.5	60	-	0.070	50.3	[26]
Chitosan fiber 3D-network	5	600	0.748	0.827	223	[27]
Phosphonic acid functionalized cellulose	5	35	2.63	3.00	100	[28]
Amidoxime-functionalized silica microspheres	5	120	1.54	1.62	225	[29]
Sugar beet pulp	5	60	-	0.086	6.43	[30]
PEI-modified p(GA-EGMA) microbeads	6	120	0.369	0.373	59.0	[31]
Trimesoyl chloride/Melamine/Palygorskite	6	75	0.588	1.16	0.95	[32]
Citric acid-treated *Luffa cylindrical* fibers	6	180	0.756	0.858	2.78	[33]
Nano-hydroxyapatite/Activated carbon/Alginate	6	420	0.044	0.078	0.95	[34]
Temperature sensitive urea-formaldehyde resin	6	300	0.399	0.416	928	[35]
Carboxymethyl konjac glucomannan/gellan gum	6	720	0.414	0.407	45.7	[36]
Bis-amidoxime functionalized marine fungus	6	110	1.56	1.56	0.38	[37]
Diethylenetriamine tethered mesoporous silica	6	180	3.87	4.48	3.95	[38]
Yarrowia lipolytica /alginate beads	7.5	60	-	0.102	2.86	[39]

Table S7. Composition of selected ore (percentage for major compounds, ppm for minors).

Compound	Percentage (%)	Metal ion	Content (ppm)
SiO_2	36.81	U	312.57
Al_2O_3	11.97	Cu	215.49
Fe_2O_3	5.33	Ni	98
CaO	3.96	V	105.01
Na_2O	3.31	Zn	735.3
Cl	3.27	Co	37.77
MgO	4.02	U	312.57
K_2O	0.54		
MnO	3.27		
L. O. I	25.88		

L.O.I.: loss on ignition.

Table S8. Composition of WPS (concentrations, mg L^{-1}; natural pH: 6.3) and sorption efficiency (%) at different pH values (using PGG@C and PGG@MC).

Element	WPS		PGG@M			PGG@MC		
			pH_{eq}			pH_{eq}		
	(mg L^{-1})	(µmol L^{-1})	2.31	3.98	5.94	2.29	3.85	5.88
Si(IV)	9.957	354.5	10.2	32.1	39.3	9.5	24.5	38.4
Al(III)	91.12	3377	2.7	12.8	16.0	1.5	10.7	13.6
Fe(III)	69.05	1236	15.1	28.4	31.8	16.2	29.1	34.0
Ca(II)	143.86	3589	5.0	23.1	25.8	5.5	22.1	29.0
Mn(II)	28.928	526.6	3.4	12.1	12.9	6.9	14.1	17.1
Ni(II)	2.03	34.59	2.0	16.7	27.1	6.4	14.3	35.0
Cu(II)	32.75	515.4	11.9	30.8	37.2	7.4	22.0	26.9
Zn(II)	9.05	85.92	10.9	41.5	43.4	11.4	33.8	44.7
U(VI)	5.62	38.03	29.8	81.3	88.9	20.7	67.7	83.3

Table S9. Ionic properties of selected elements (hydrated)

**Element**	**Structure (a)**	**Solution-phase electronegativity (b)**	**Hydrated radius (Å) (a)**	**Hydrated radius (Å) (c)**	**-ΔG° (kcal mol^{-1}) (b)**	**Soft-ness σ (d)**
Si(IV)	-	-	-	-	-	
Al(III)	$Al(H_2O)_6^{3+}$	3.435	0.535	-	1081.5	-0.31
Fe(III)	$Fe(H_2O)_6^{3+}$	3.835	0.645	-	1019.4	0.33
Mg(II)	$Mg(H_2O)_6^{2+}$	2.158	0.72	0.72	437.4	-0.41
Ca(II)	$Ca(H_2O)_8^{2+}$	1.862	1.12	1.00	359.7	-0.66
Mn(II)	$Mn(H_2O)_6^{2+}$	2.458	0.830	0.82	420.7	-0.15
Ni(II)	$Ni(H_2O)_6^{2+}$	2.891	0.690	0.70	473.2	-0.11
Cu(II)	$Cu(H_2O)_6^{2+}$	2.952	0.73	0.73	480.4	0.38
Zn(II)	$Zn(H_2O)_6^{2+}$	2.796	0.74	0.75	467.3	0.35
Nd(III)	$Nd(H_2O)_9^{3+}$	3.085	1.16	1.16	783.9	-0.58
U(VI)	$UO_2(H_2O)_5^{2+}$	-	1.08	0.75	*	-0.27

(a): [40]; (b): [41]; (c): [42]; (d): [43]. ;*: strongly depends on the speciation of uranyl [44].

Xu et al [42] scaled the metal ions in function of an acid softness index expressed as the Gibbs free energy of formation:

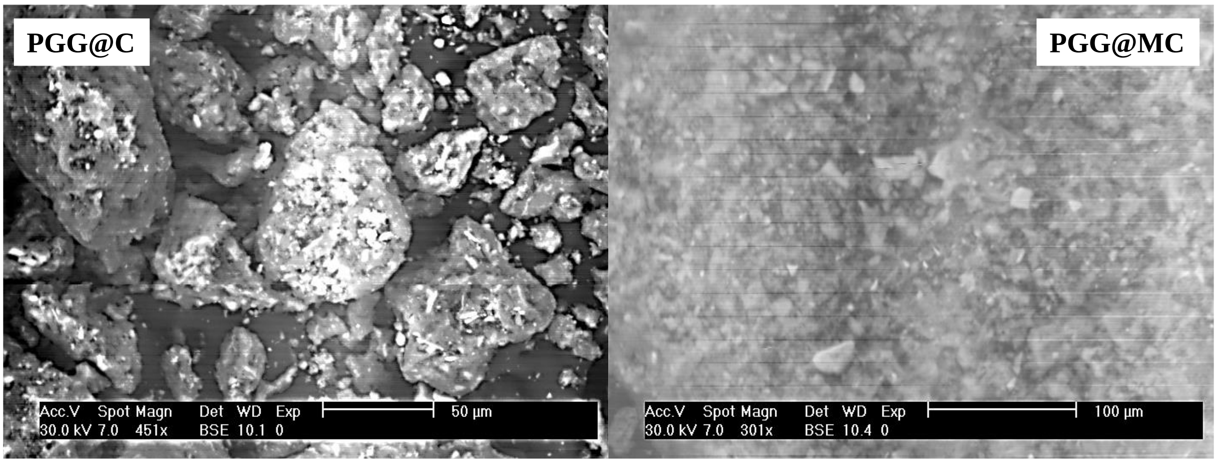

Figure S1. SEM images of PGG@C and PGG@MC.

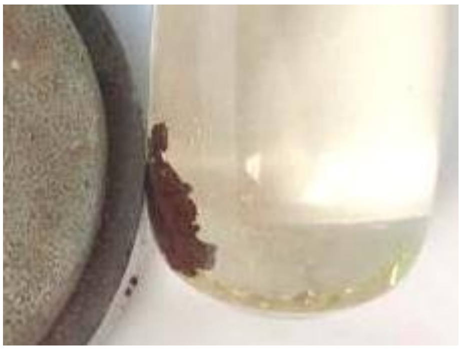

Magnetic separation

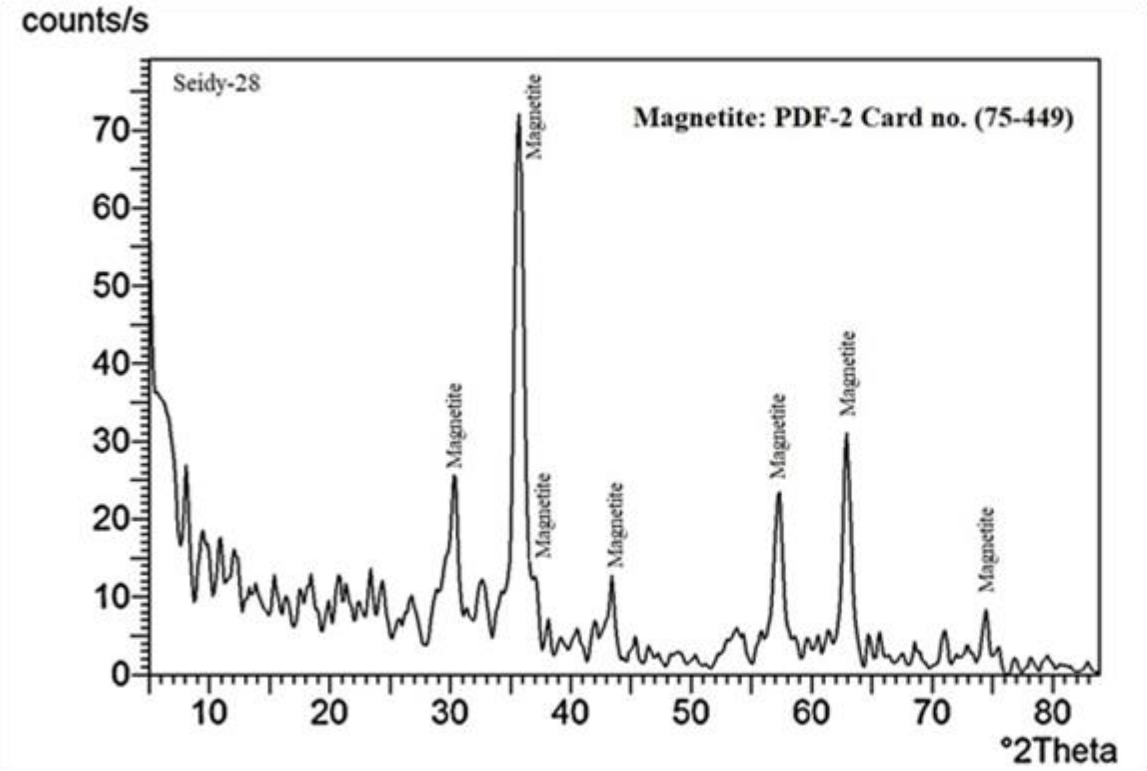

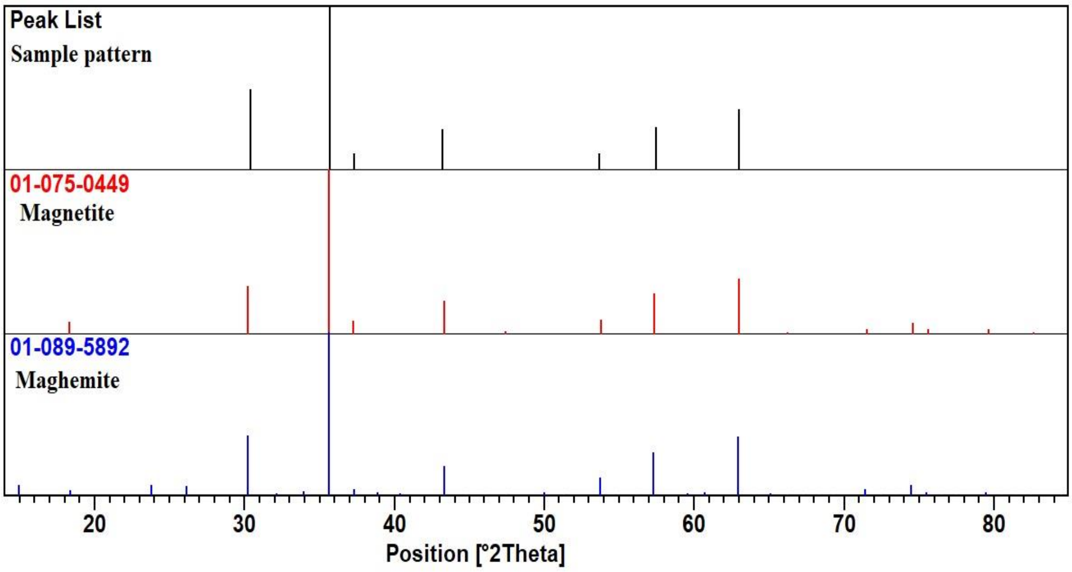

Sample data			Crystal planes	01-075-0449 Magnetite, Fe_3O_4		01-089-5892 Maghemite	
**Position [2θ, deg.]**	**d-spacing [Å]**	**Rel. Int. [%]**		**d-spacing [Å]**	**Rel. Int. [%]**	**d-spacing [Å]**	**Rel. Int. [%]**
30.3268	2.94731	35.2	(220)	2.94	28	2.95	36
35.6527	2.5183	100	(311)	2.50	100	2.50	100
37.3	2.41	9.0	(222)	2.41	7	2.40	3
43.27	2.091	15.19	(400)	2.08	19	2.08	17
57.5013	1.60278	15.19	(511)	1.60	23	1.6	26
63.0824	1.47375	40.89	(440)	1.47	33	1.47	35
74.4108	1.27496	15.62	(533)	1.26	6	1.27	6

XRD analysis

Figure S2. Structural characterization of PGG@MC (magnetic behavior) and XRD diffraction patterns.

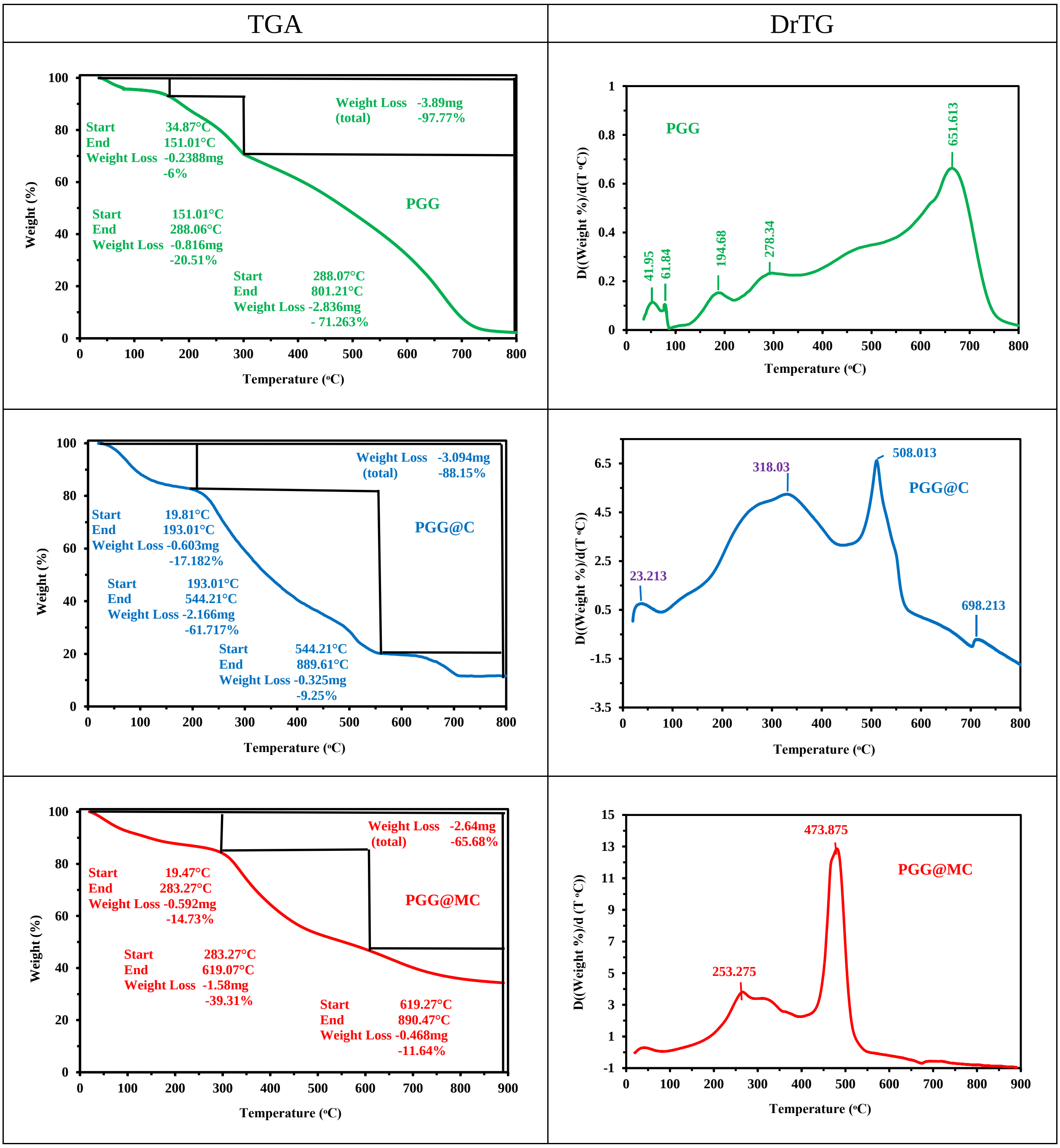

Figure S3. Thermogravimetric analysis (TGA and DrTG) of PGG, PGG@C and PGG@MC.

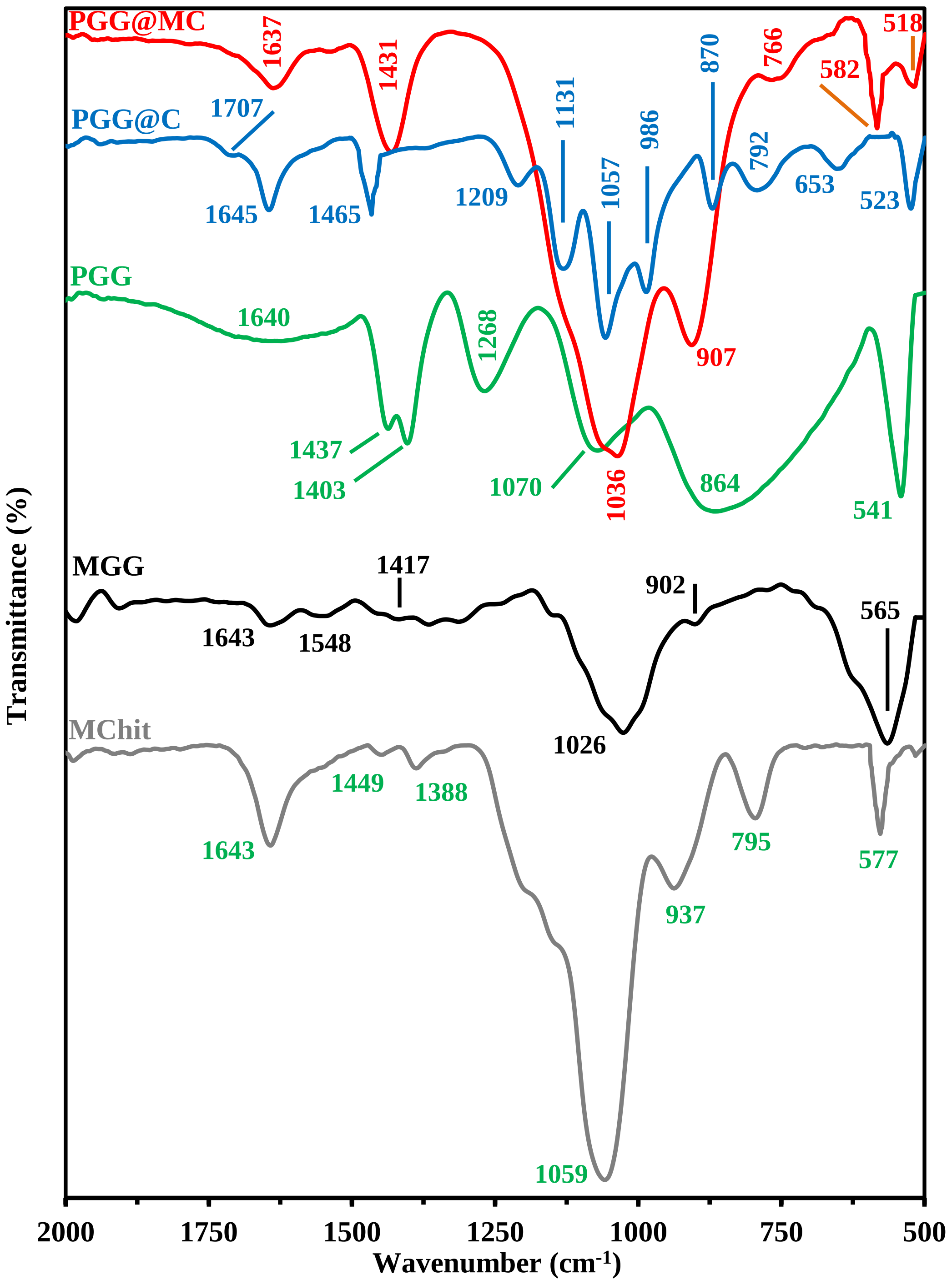

Figure S4. FTIR spectra of different functionalized materials and composites (wavenumber range: 2000-500 cm^{-1}).

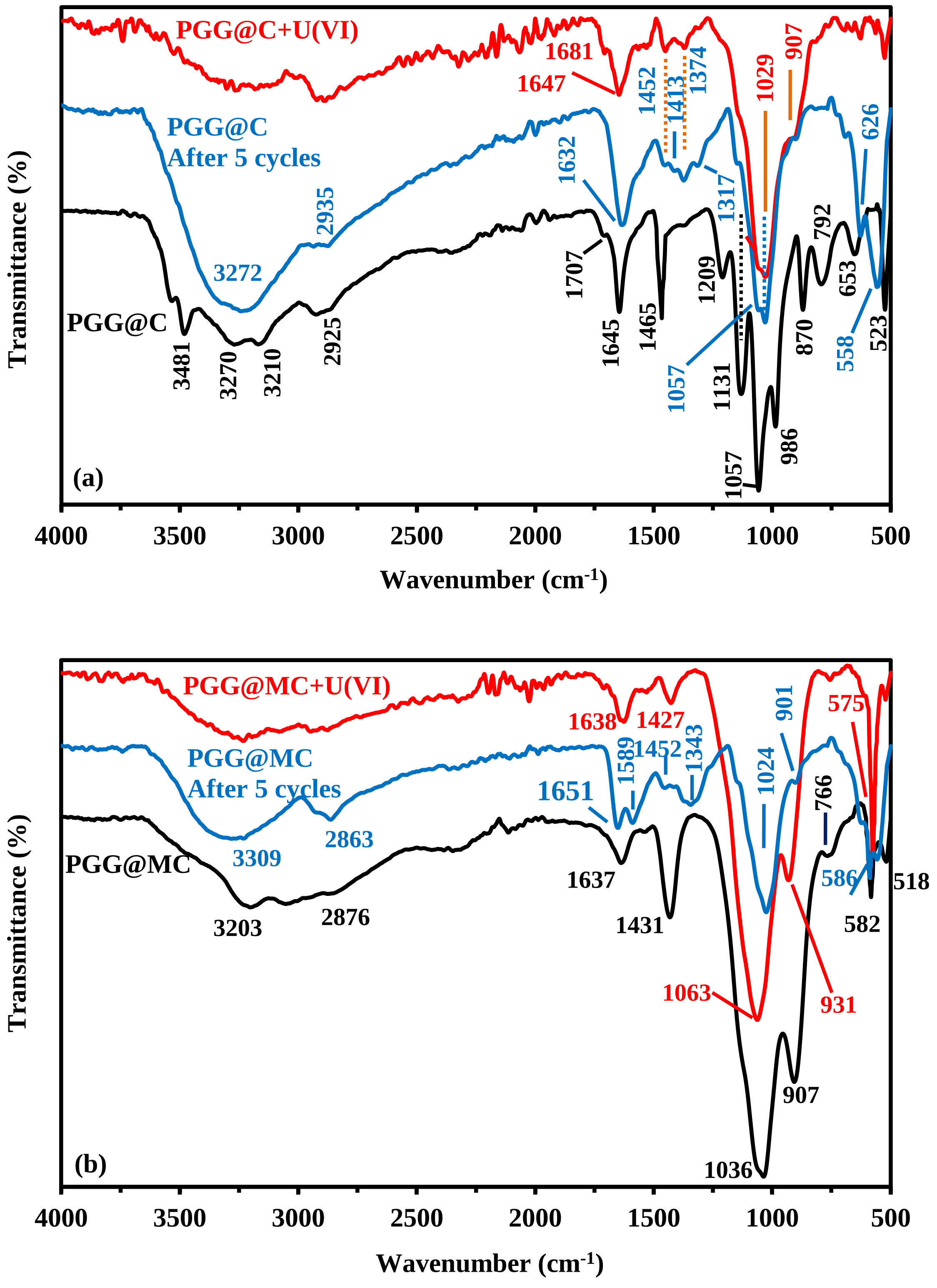

Figure S5. FTIR spectra of PGG@C (a) and PGG@MC (b), after U(VI) sorption and after 5 cycles of recycling.

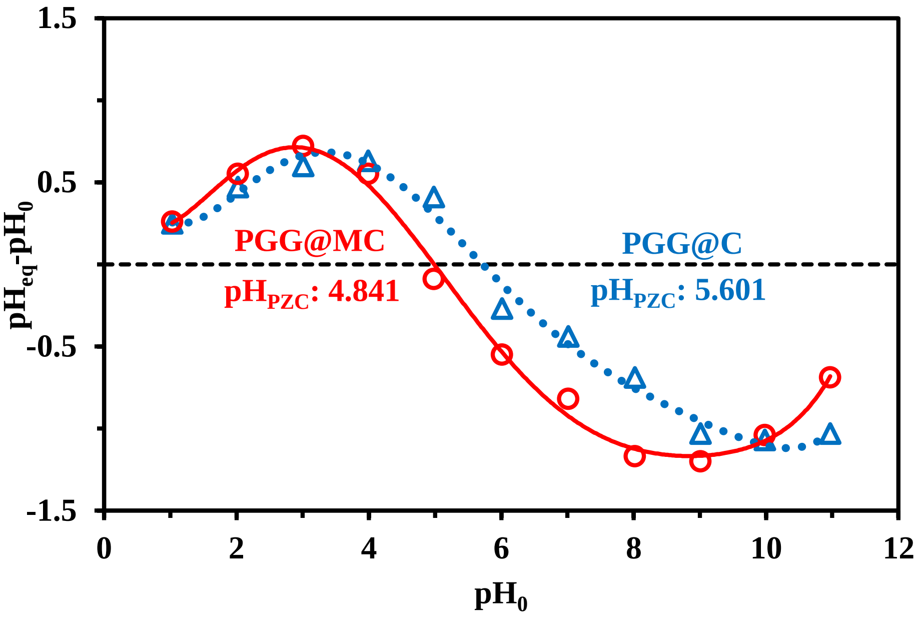

Figure S6. Determination of pH_{PZC} of PGG@C and PGG@MC sorbents (Sorbent dosage, SD: 2 g L^{-1}; Background salt: 0.1 M; Time: 48 h).

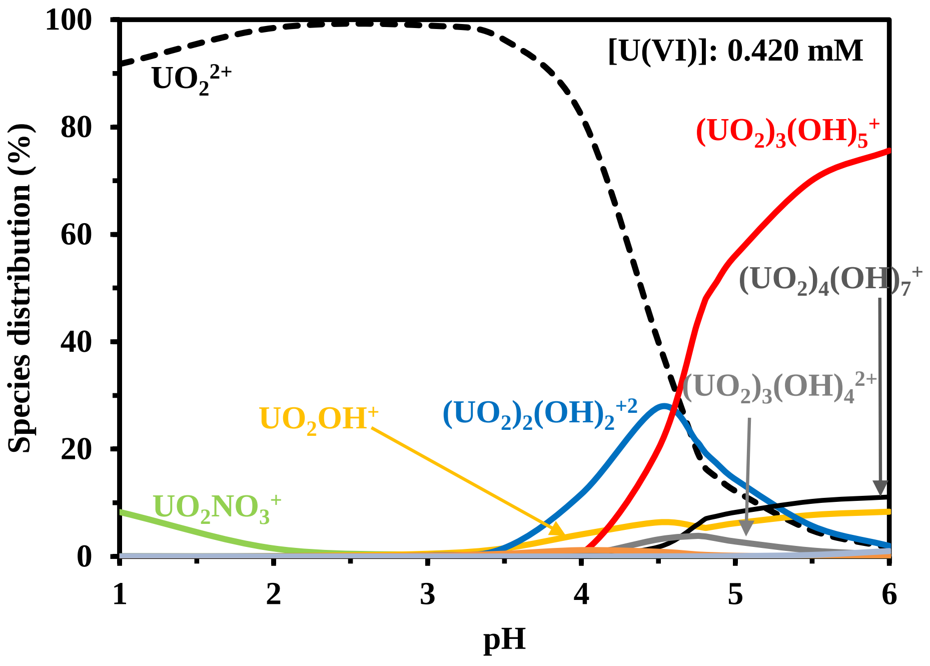

Figure S7. U(VI) speciation – Effect of pH (experimental conditions corresponding to the study of pH effect - C_0: 0.42 mmol U L^{-1}; pH controlled with HNO_3 or NaOH; precipitation begins above pH 4.8; calculated using Visual Minteq, [45]).

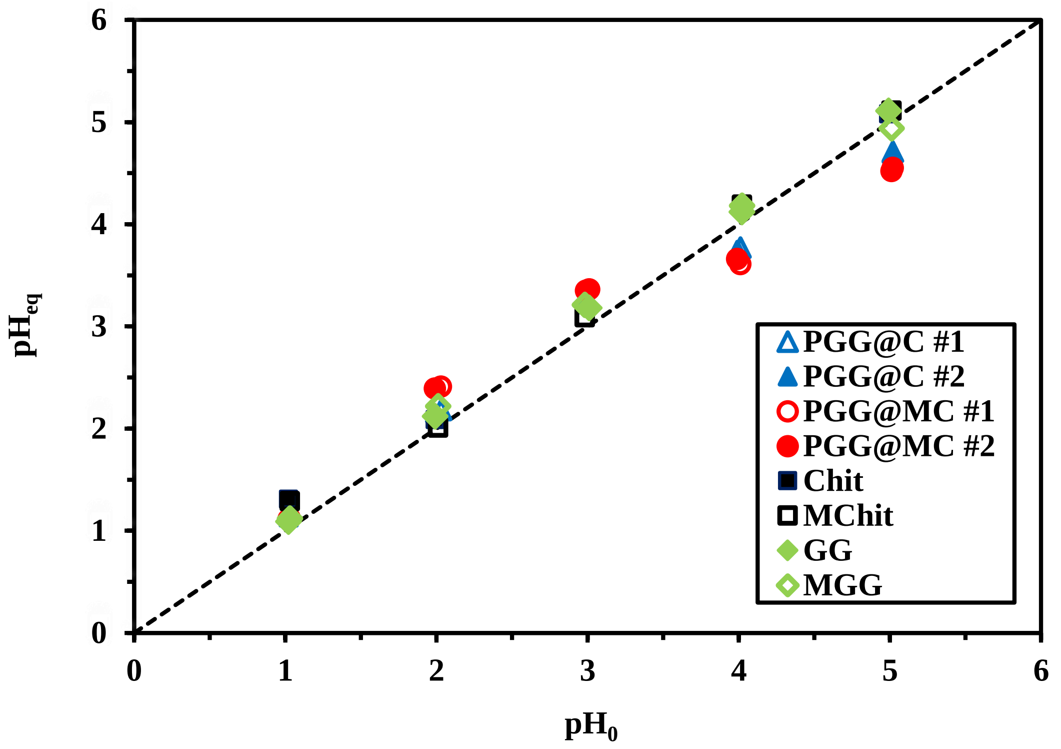

Figure S8. pH variation during U(VI) sorption using PGG@C and PGG@MC (and intermediary products: Chit, MChit, GG, and MGG) (C_0: 0.42 mmol U L^{-1}; Sorbent dosage, SD: 833 mg L^{-1}; Time: 48 h; v: 200 rpm; T: 21 ±1 °C).

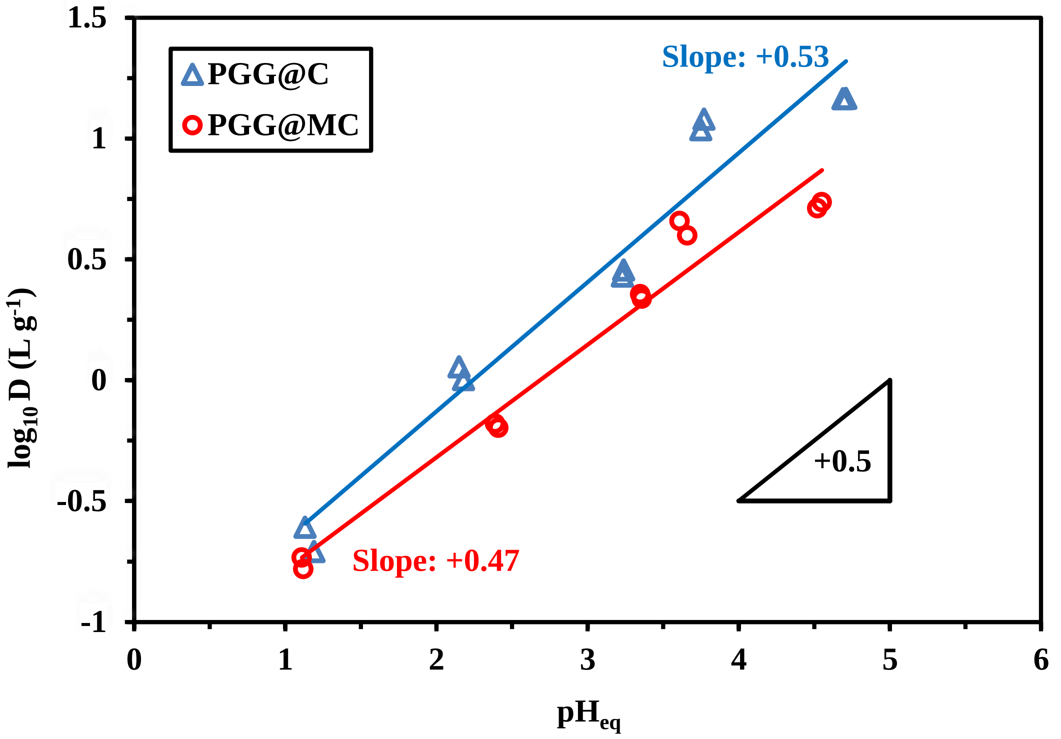

Figure S9. Plot of $\log_{10}$ D (L g^{-1}) *vs.* pH_{eq} for U(VI) sorption using PGG@C and PGG@MC.

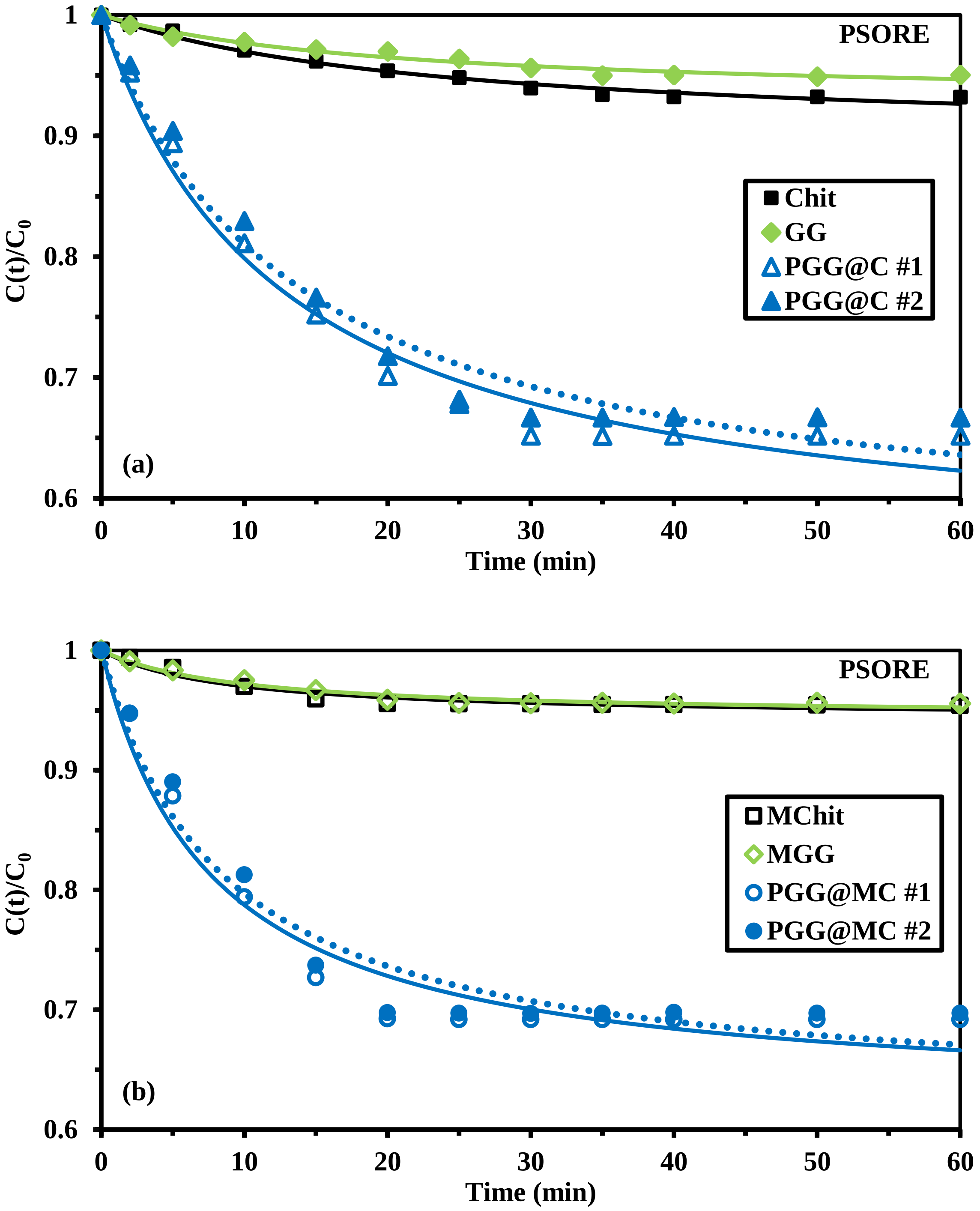

Figure S10. U(VI) uptake kinetics using Chit, GG, and PGG@C sorbents: non-magnetic (a) and magnetic (b) sorbents – Modeling with the PSORE (C_0: 0.42 mmol U L^{-1}; pH_0: 4; Sorbent dosage, SD: 300 mg L^{-1}; v: 200 rpm; T: 21 ±1 °C).

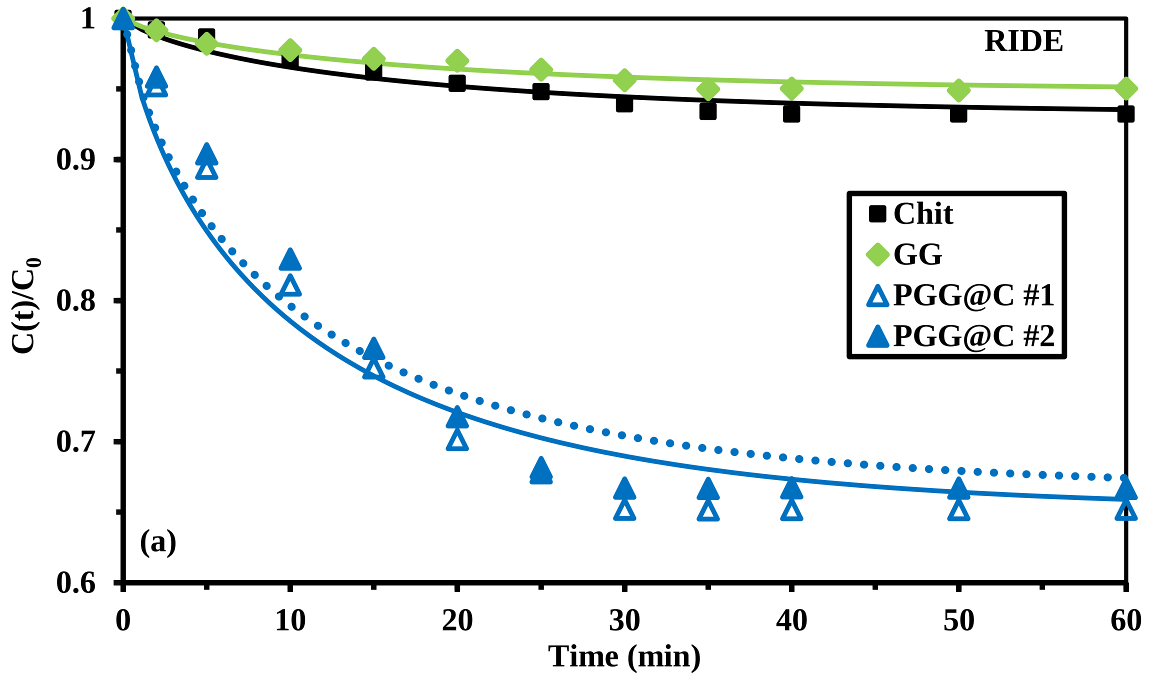

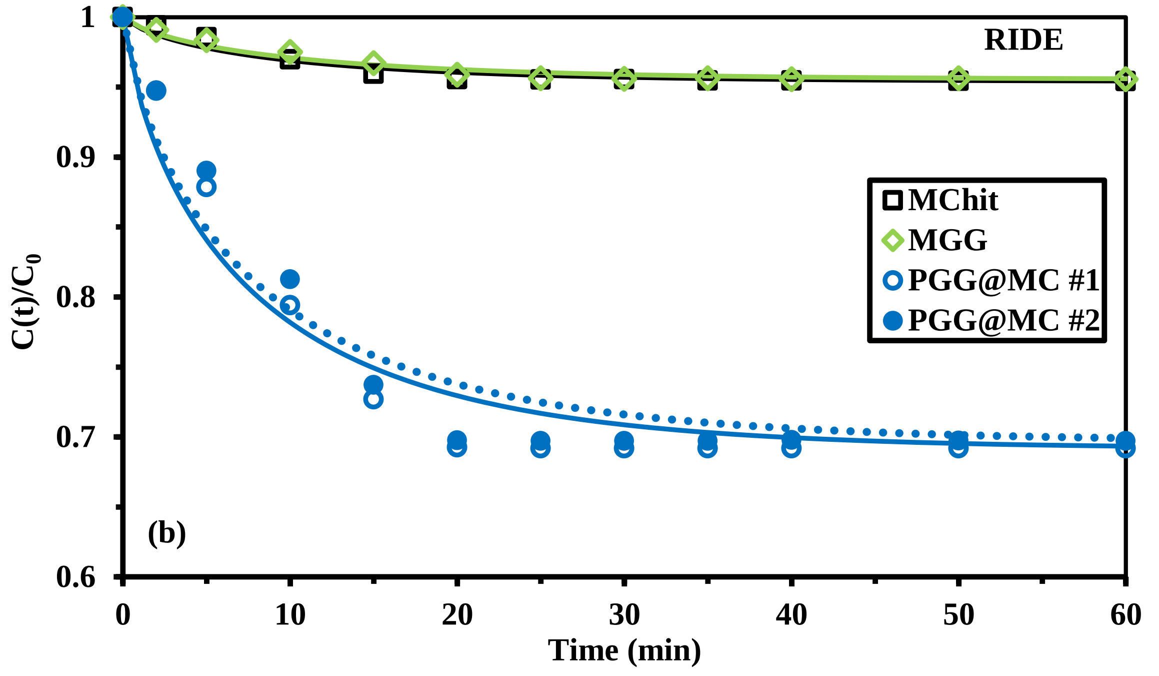

Figure S11. U(VI) uptake kinetics using Chit, GG, and PGG@C sorbents: non-magnetic (a) and magnetic (b) sorbents – Modeling with the PSORE (C_0: 0.42 mmol U L^{-1}; pH_0: 4; Sorbent dosage, SD: 300 mg L^{-1}; v: 200 rpm; T: 21 ±1 °C).

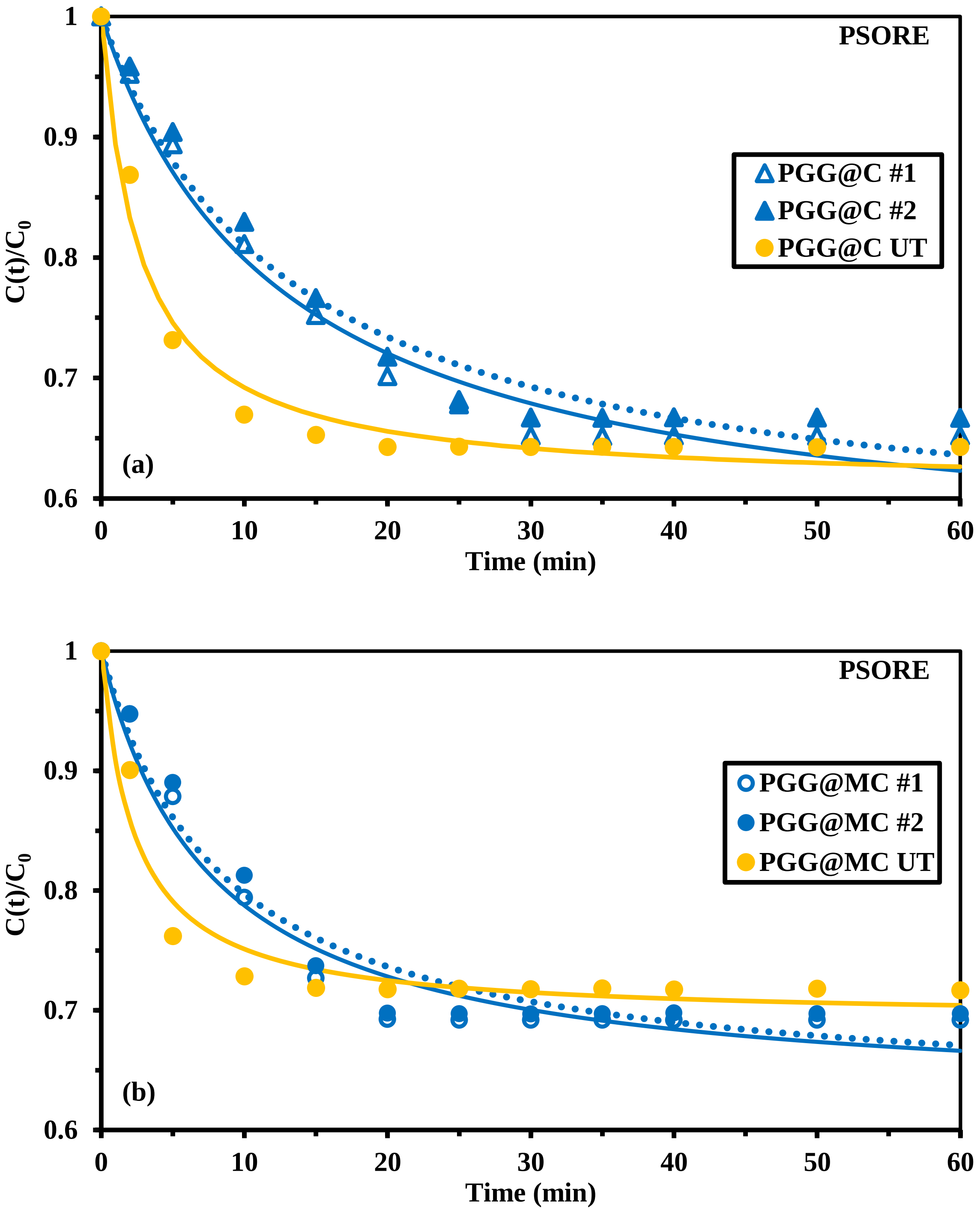

Figure S12. Effect of ultrasonic treatment (UT) on U(VI) uptake kinetics using PGG@C (a) and PGG@MC (b) sorbents – Modeling with the PSORE (C_0: 0.42 mmol U L^{-1}; pH_0: 4; Sorbent dosage, SD: 300 mg L^{-1}; v: 200 rpm; T: 21 ±1 °C).

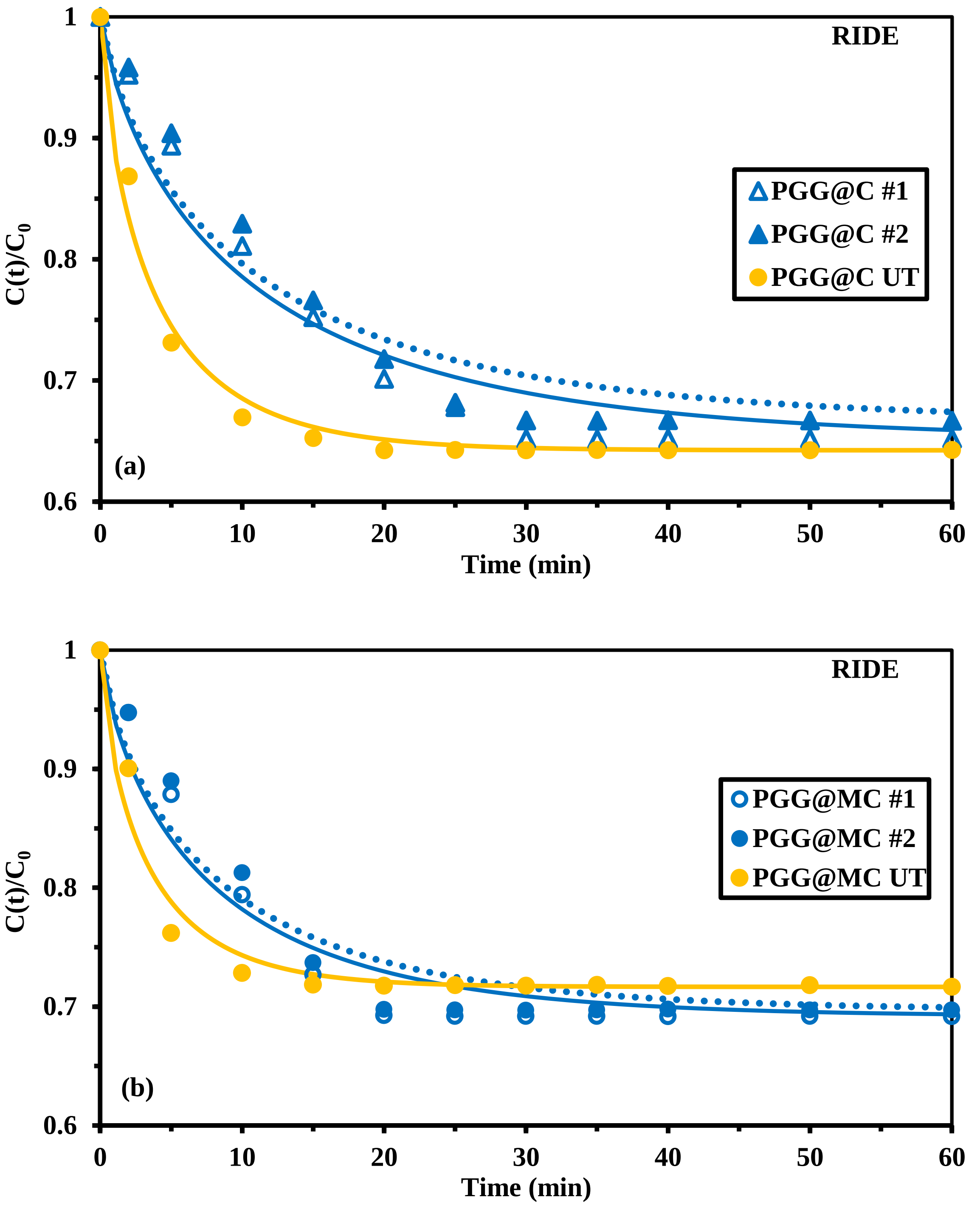

Figure S13. Effect of ultrasonic treatment (UT) on U(VI) uptake kinetics using PGG@C (a) and PGG@MC (b) sorbents – Modeling with the RIDE (C_0: 0.42 mmol U L^{-1}; pH_0: 4; Sorbent dosage, SD: 300 mg L^{-1}; v: 200 rpm; T: 21 ±1 °C).

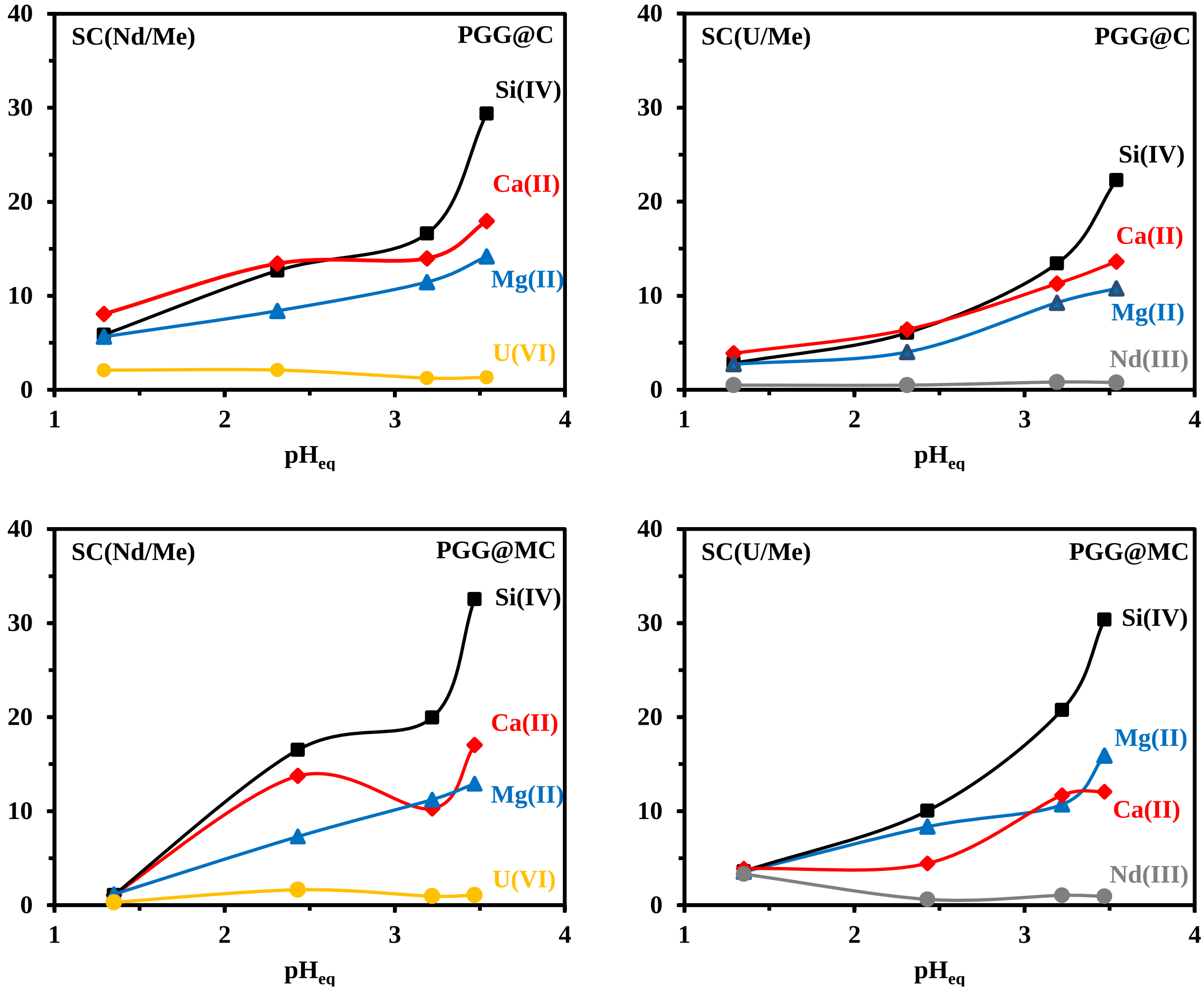

Figure S14. Effect of pH on the selectivity coefficients $SC_{(Nd/Me)}$ and $SC_{(U/Me)}$ for PGG@C and PGG@MC (C_0: 0.5 mmol L^{-1}; SD: 833 mg L^{-1}; v: 200 rpm; T: 21 ±1 °C; Time: 10 h).

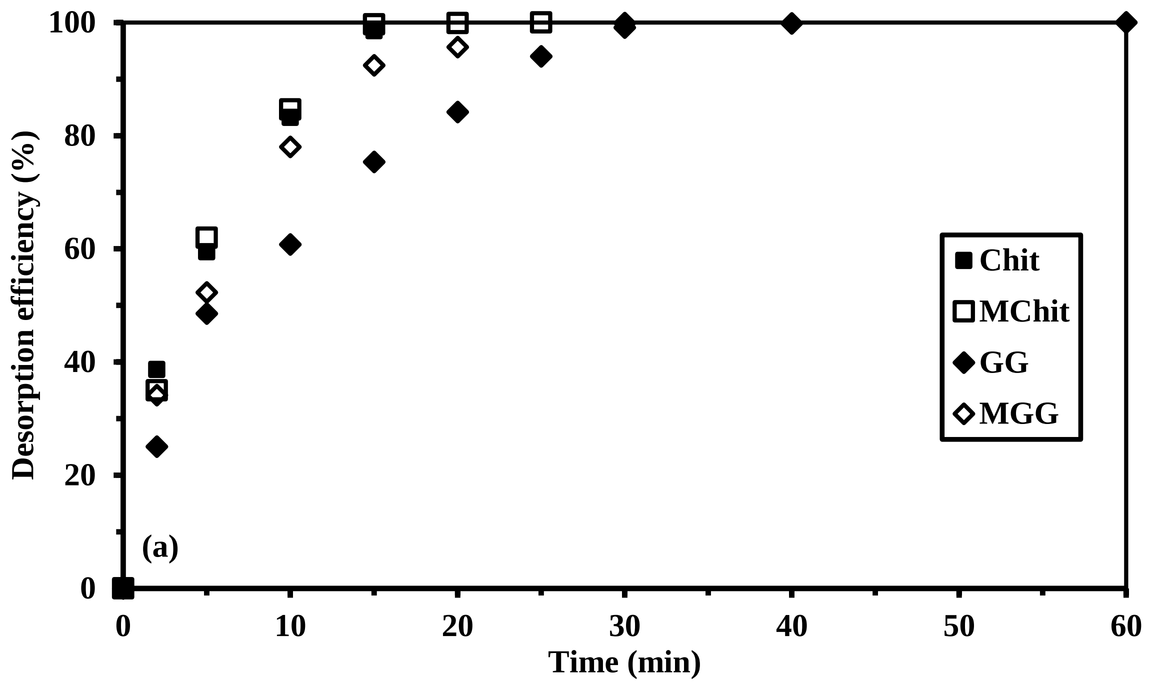

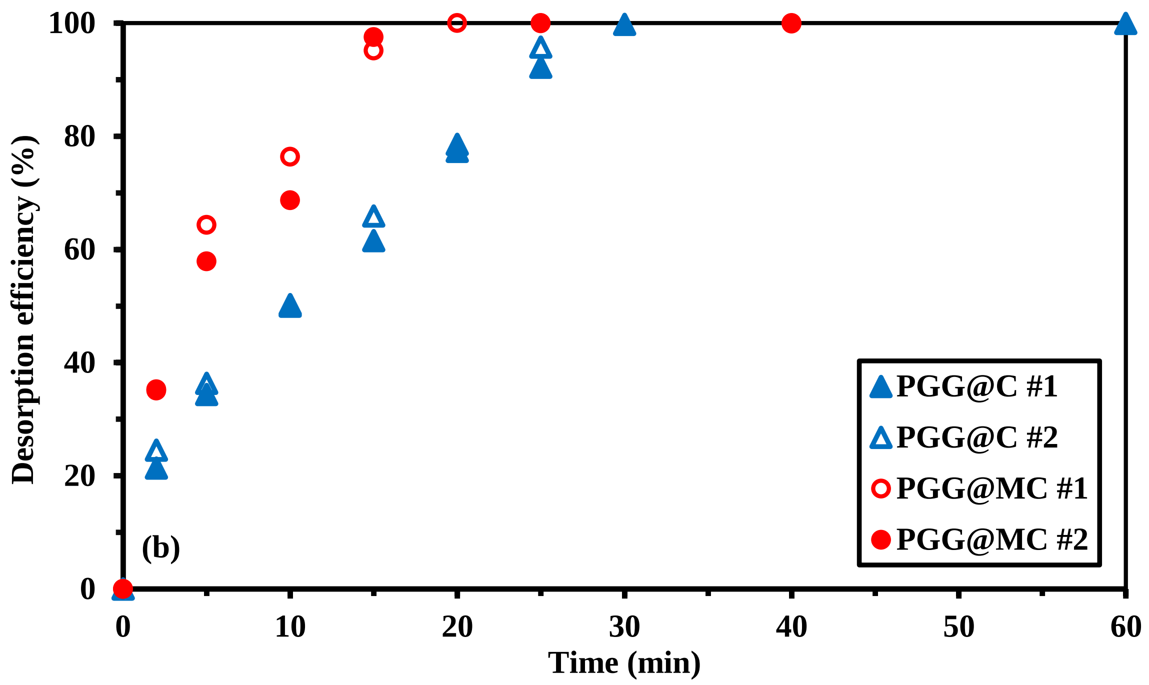

Figure S15. Comparison of U(VI) desorption kinetics for selected sorbents (metal-loaded sorbents collected from uptake kinetics; 0.2 M HCl eluent; SD: 1.2 g L^{-1}; v: 200 rpm; T: 21 ±1 °C).

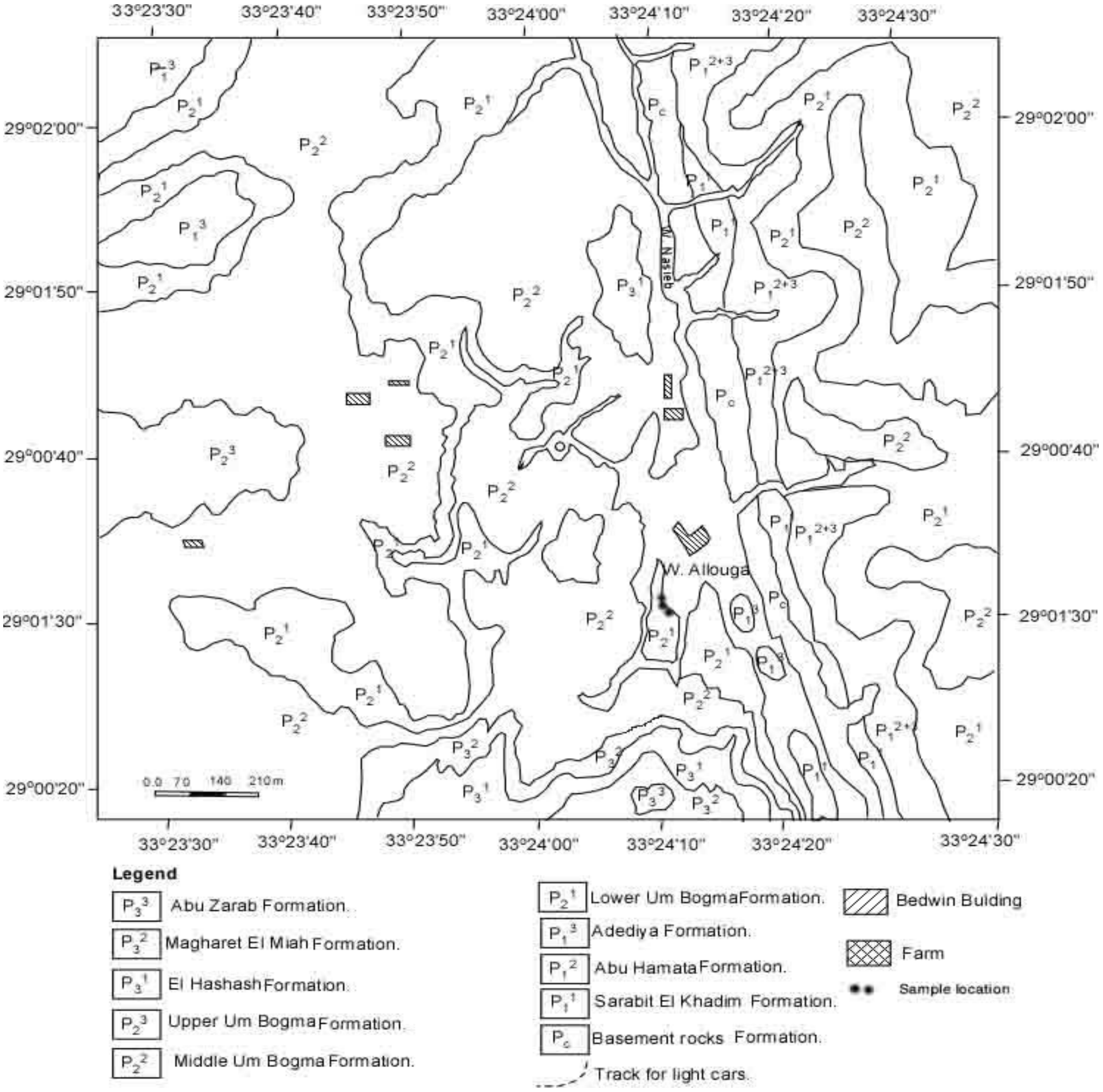

Figure S16. Geological and location map of mining area.

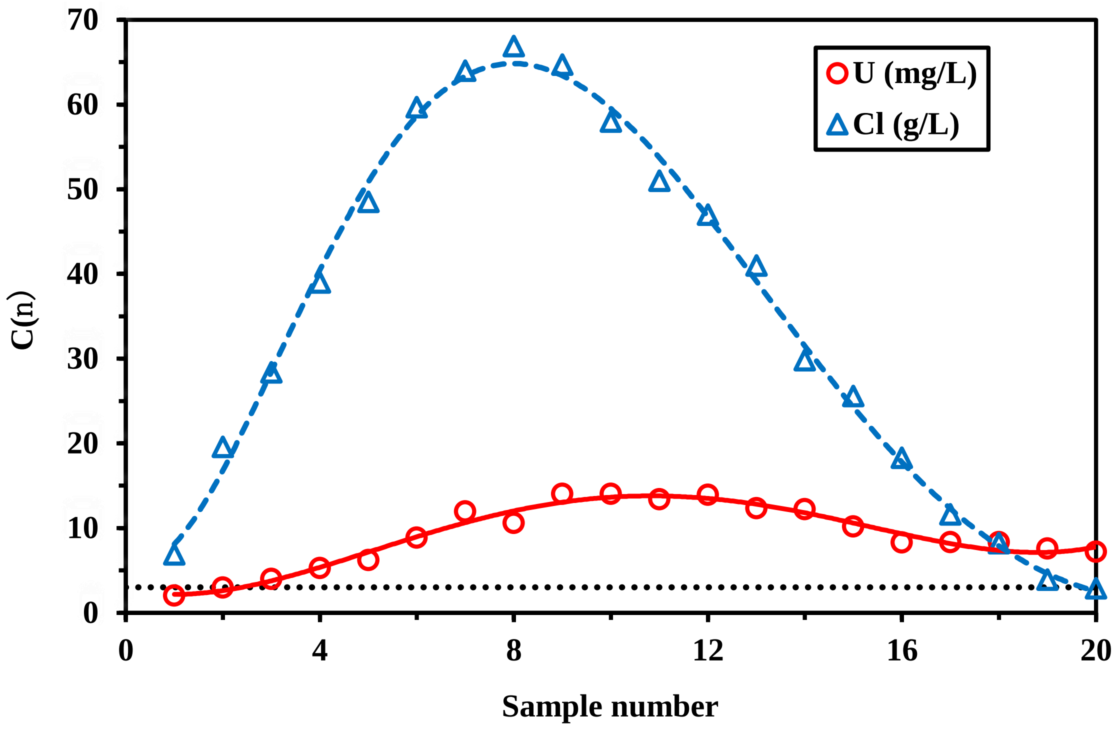

Figure S17. Water washing of ore – Evolution of the concentrations of Cl^- (g L^{-1}) and U(VI) (mg L^{-1}) at the outlet of the column (Volume fractions: 20 mL; dot line: outlet Cl^- concentration: 3 g L^{-1}).

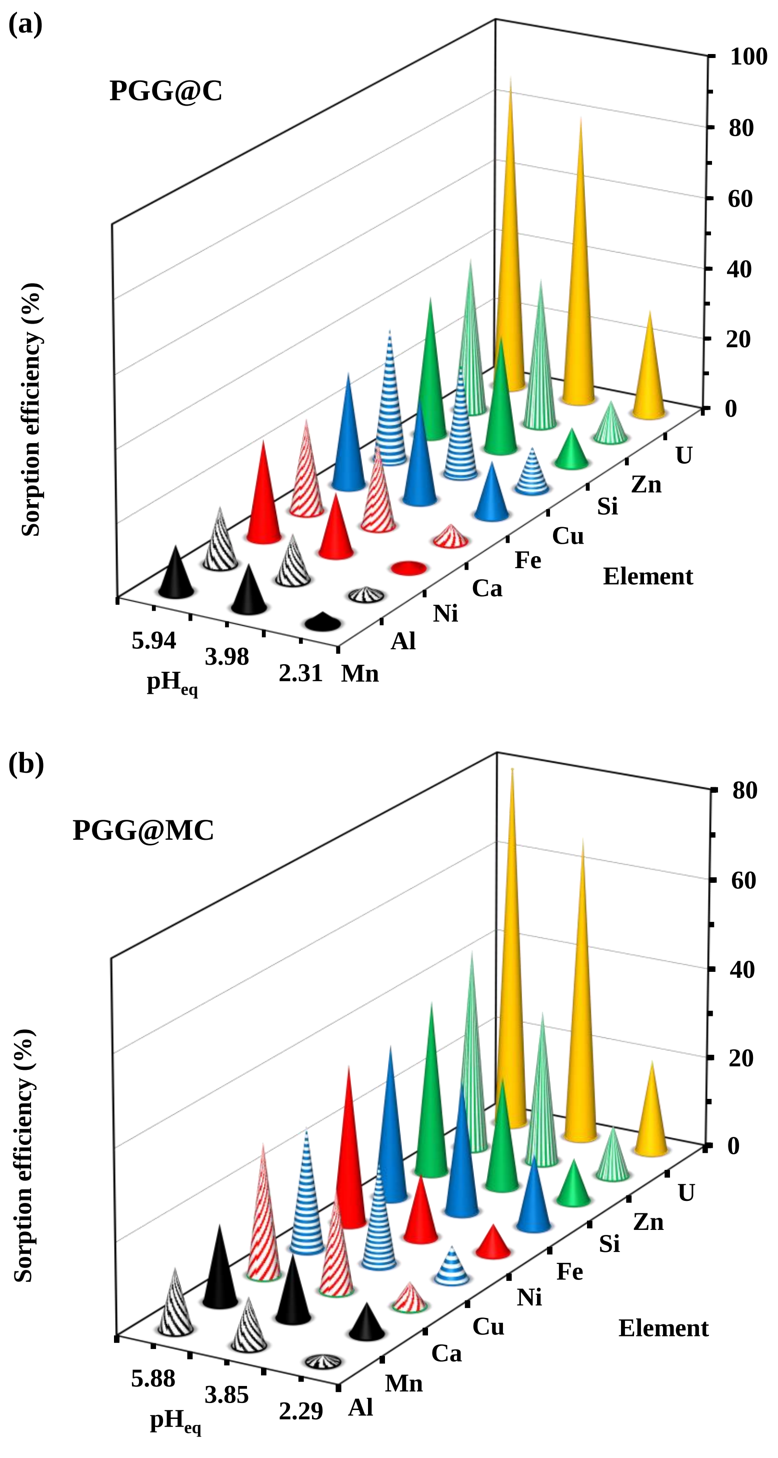

Figure S18. Effect of pH_{eq} on sorption efficiency (%) for target elements from WPS using PGG@C and PGG@MC.

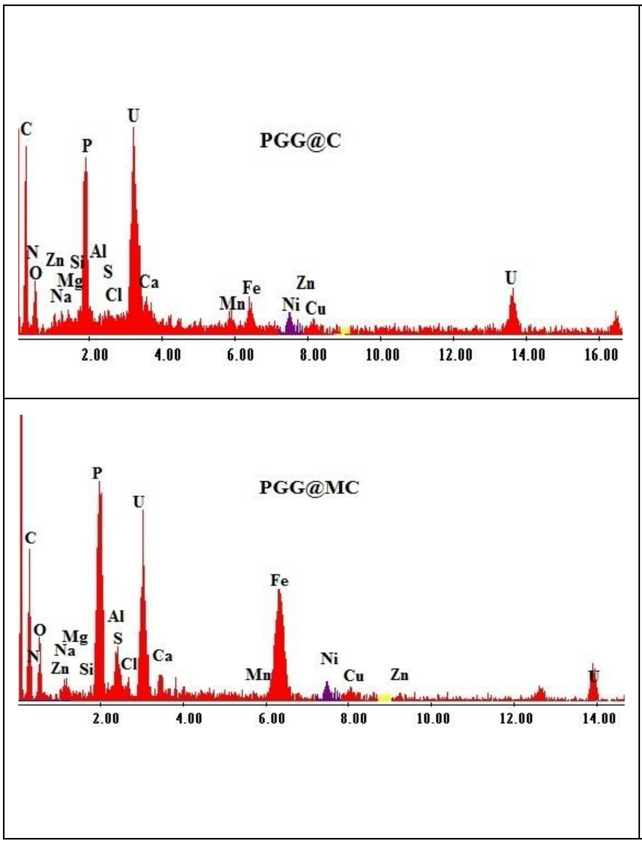

Element	PGG@C		PGG@MC	
	At. (%)	Wt. (%)	At. (%)	Wt. (%)
C	36.8	29.22	31.64	26.29
O	32.96	27.83	34.85	33.54
N	5.81	7.84	4.17	5.35
P	8.95	9.81	6.54	5.43
Cl	1.29	1.75	0.97	2.04
S	1.03	3.05	0.47	3.24
Si	1.36	1.95	1.01	1.38
Na	1.11	2.47	0.89	3.01
Al	0.67	1.03	0.79	1.11
Fe	0.1	0.93	12.09	9.02
Ca	0.74	1.94	0.52	2.03
Mg	0.83	0.51	0.41	0.36
Mn	0.32	0.53	0.29	0.31
Cu	0.03	0.1	0.01	0.09
Ni	0.09	0.1	0.01	0.11
Zn	0.12	0.85	0.01	0.13
U	7.79	10.09	5.33	6.56

Figure S19. Semi-quantitative EDX analyses of PCC@C and PGG@MC after contact with WPS.

References

1. Ho, Y. S.; McKay, G., Pseudo-second order model for sorption processes. *Process Biochem.* **1999,** 34, (5), 451-465.
2. Tien, C., *Adsorption Calculations and Modeling*. Butterworth-Heinemann: Newton, MA, 1994 p 243.
3. Langmuir, I., The adsorption of gases on plane surfaces of glass, mica and platinum. *J. Amer. Chem. Soc.* **1918,** 40, 1361-1402.
4. Freundlich, H. M. F., Uber die adsorption in lasungen. *Z. Phys. Chem.* **1906,** 57, 385-470.
5. Nayak, A. K.; Pal, A., Development and validation of an adsorption kinetic model at solid-liquid interface using normalized Gudermannian function. *J. Mol. Liq.* **2019,** 276, 67-77.
6. Venkatesan, K. A.; Shyamala, K. V.; Antony, M. P.; Srinivasan, T. G.; Rao, P. R. V., Batch and dynamic extraction of uranium(VI) from nitric acid medium by commercial phosphinic acid resin, Tulsion CH-96. *J. Radioanal. Nucl. Chem.* **2008,** 275, (3), 563-570.
7. Zhang, S.; Yuan, D.; Zhang, Q.; Wang, Y.; Liu, Y.; Zhao, J.; Chen, B., Highly efficient removal of uranium from highly acidic media achieved using a phosphine oxide and amino functionalized superparamagnetic composite polymer adsorbent. *J. Mater. Chem. A* **2020,** 8, (21), 10925-10934.
8. Mosleh, M. A.; El-Hakim, E. H.; Ahmed, A. Z.; Abd El-Ghany, M. S.; El-Didamony, A. M., Equilibrium and kinetic studies on uranium sorption from aqueous sulphate medium using tri-n-octylamine impregnated resin. *Int. J. Environ. Anal. Chem.* **2020**, Art. N° 1766038.
9. Wen, Z.; Huang, K.; Niu, Y.; Yao, Y.; Wang, S.; Cao, Z.; Zhong, H., Kinetic study of ultrasonic-assisted uranium adsorption by anion exchange resin. *Colloids Surf., A* **2020,** 585, Art. N° 124021.
10. Masoud, A. M., Sorption behavior of uranium from sulfate media using purolite A400 as a strong base anion exchange resin. *Int. J. Environ. Anal. Chem.* **2020**, Doi: 10.1080/03067319.2020.1763974.
11. Ahmad, A. A., Kinetics of uranium adsorption from sulfate medium by a commercial anion exchanger modified with quinoline and silicate. *J. Radioanal. Nucl. Chem.* **2020,** 324, (3), 1387-1403.
12. Zidan, I. H.; Cheira, M. F.; Bakry, A. R.; Atia, B. M., Potentiality of uranium recovery from G.Gattar leach liquor using Duolite ES-467 chelating resin: Kinetic, thermodynamic and isotherm features. *Int. J. Environ. Anal. Chem.* **2020**, Art. N° 1748613.

13. Yousef, L. A.; Ahmad, A. A.; Bakry, A. R., Separation of uranium ions from acetate medium by Dowex50WX8/Alizarin Red-S and its application on granitic samples, South Um Tawat, Eastern Desert. *Int. J. Environ. Anal. Chem.* **2020**, Doi: 10.1080/03067319.2020.1772771.
14. Solgy, M.; Taghizadeh, M.; Ghoddocynejad, D., Adsorption of uranium(VI) from sulphate solutions using Amberlite IRA-402 resin: Equilibrium, kinetics and thermodynamics study. *Ann. Nucl. Energy* **2015,** 75, 132-138.
15. Semnani, F.; Asadi, Z.; Samadfam, M.; Sepehrian, H., Uranium(VI) sorption behavior onto amberlite CG-400 anion exchange resin: Effects of pH, contact time, temperature and presence of phosphate. *Ann. Nucl. Energy* **2012,** 48, 21-24.
16. Yuvaraja, G.; Su, M.; Chen, D.-Y.; Pang, Y.; Kong, L.-J.; Subbaiah, M. V.; Wen, J.-C.; Reddy, G. M., Impregnation of magnetic - *Momordica charantia* leaf powder into chitosan for the removal of U(VI) from aqueous and polluted wastewater. *Int. J. Biol. Macromol.* **2020,** 149, 127-139.
17. Yuan, Y.; Liu, N.; Dai, Y.; Wang, B.; Liu, Y.; Chen, C.; Huang, D., Effective biosorption of uranium from aqueous solution by cyanobacterium *Anabaena flos-aquae*. *Environ. Sci. Pollut. Res.* **2020,** 27, 44306-44313.
18. Yousef, L. A.; Bakry, A. R.; Ahmad, A. A., Uranium(VI) recovery from acidic leach liquor using manganese oxide coated zeolite (MOCZ) modified with amine. *J. Radioanal. Nucl. Chem.* **2020,** 324, (1), 409-421.
19. Yang, J.-h.; Lei, Z.-j.; Dai, Y.-h.; Luo, Y.; Xie, S.-b.; Wang, J.-s.; Zhou, S.-k.; Wei, B.; Li, C.; Hu, S.-q., Preparation of aluminum sludge composite gel spheres and adsorption of U(IV) from aqueous solution. *Environ. Sci. Pollut. Res.* **2020,** 27, (21), 26835-26844.
20. Zhang, M.; Yuan, M.; Zhang, M.; Wang, M.; Chen, J.; Li, R.; Qiu, L.; Feng, X.; Hu, J.; Wu, G., Efficient removal of uranium from diluted aqueous solution with hydroxypyridone functionalized polyethylene nonwoven fabrics. *Radiat. Phys. Chem.* **2020,** 171, Art. N° 108742.
21. Rashad, M. M.; El-Sayed, I. E.; Galhoum, A. A.; Abdeen, M. M.; Mira, H. I.; Elshehy, E. A.; Zhang, S.; Lu, X.; Xin, J.; Guibal, E., Synthesis of α-aminophosphonate based sorbents – Influence of inserted groups (carboxylic vs. amine) on uranyl sorption. *Chem. Eng. J.* **2020**, Art. N° 127830.
22. Galhoum, A. A.; Eisa, W. H.; El-Sayed, I. E.-T.; Tolba, A. A.; Shalaby, Z. M.; Mohamady, S. I.; Muhammad, S. S.; Hussien, S. S.; Akashi, T.; Guibal, E., A new route for manufacturing poly(aminophosphonic)-functionalized poly(glycidyl methacrylate)-magnetic nanocomposite - Application to uranium sorption from ore leachate. *Environ. Pollut* **2020,** 264, Art. N° 114797.
23. Hamza, M. F.; Roux, J.-C.; Guibal, E., Uranium and europium sorption on amidoxime-functionalized magnetic chitosan micro-particles. *Chem. Eng. J.* **2018,** 344, 124-137.
24. Imam, E. A.; El-Tantawy El-Sayed, I.; Mahfouz, M. G.; Tolba, A. A.; Akashi, T.; Galhoum, A. A.; Guibal, E., Synthesis of α-aminophosphonate functionalized chitosan sorbents: Effect of methyl vs phenyl group on uranium sorption. *Chem. Eng. J.* **2018,** 352, 1022-1034.
25. Cheira, M. F.; Kouraim, M. N.; Zidan, I. H.; Mohamed, W. S.; Hassanein, T. F., Adsorption of U(VI) from sulfate solution using montmorillonite/polyamide and nano-titanium oxide/polyamide nanocomposites. *J. Environ. Chem. Eng.* **2020,** 8, (5), Art. N°104427.
26. Liu, H.-J.; Jing, P.-F.; Liu, X.-Y.; Du, K.-J.; Sun, Y.-K., Synthesis of beta-cyclodextrin functionalized silica gel and its application for adsorption of uranium(VI). *J. Radioanal. Nucl. Chem.* **2016,** 310, (1), 263-270.
27. Wang, Y.; Li, Y.; Li, L.; Kong, F.; Lin, S.; Wang, Z.; Li, W., Preparation of three-dimensional fiber-network chitosan films for the efficient treatment of uranium-contaminated effluents. *Water Sci. Technol.* **2020,** 81, (1), 52-61.
28. Huo, Z.; Zhao, S.; Yi, J.; Zhang, H.; Li, J., Biomass-based cellulose functionalized by phosphonic acid with high selectivity and capacity for capturing U(VI) in aqueous solution. *Appl. Sci.* **2020,** 10, (16).
29. Xiao, J.; Jing, Y.; Wang, X. Q.; Yao, Y.; Jia, Y. Z., Preconcentration of uranium(VI) from aqueous solution by amidoxime-functionalized microspheres silica material: Kinetics, isotherm and mechanism study. *Chemistryselect* **2018,** 3, (43), 12346-12356.
30. Nuhanovic, M.; Grebo, M.; Draganovic, S.; Memic, M.; Smjecanin, N., Uranium(VI) biosorption by sugar beet pulp: equilibrium, kinetic and thermodynamic studies. *J. Radioanal. Nucl. Chem.* **2019,** 322, 2065-2078.
31. Bayramoglu, G.; Arica, M. Y., Polyethylenimine and tris(2-aminoethyl)amine modified p(GA-EGMA) microbeads for sorption of uranium ions: equilibrium, kinetic and thermodynamic studies. *J. Radioanal. Nucl. Chem.* **2017,** 312, (2), 293-303.
32. Tuzen, M.; Saleh, T. A.; Sari, A.; Naeemullah, Interfacial polymerization of trimesoyl chloride with melamine and palygorskite for efficient uranium ions ultra-removal. *Chem. Eng. Res. Des.* **2020,** 159, 353-361.
33. Turanov, A. N.; Karandashev, V. K.; Emel'chenko, G. A.; Su, S.; Liu, Q.; Wang, J., Sorption of U(VI) from aqueous solutions by chemically modified *Luffa cylindrica* fibers. *Russ. J. Phys. Chem. A* **2020,** 94, (7), 1471-1475.
34. Saha, S.; Basu, H.; Rout, S.; Pimple, M. V.; Singhal, R. K., Nano-hydroxyapatite coated activated carbon impregnated alginate: A new hybrid sorbent for uranium removal from potable water. *J. Environ. Chem. Eng.* **2020,** 8, (4), Art. N° 103999.
35. Ma, D.; Wei, J.; Zhao, Y.; Chen, Y.; Tang, S., The removal of uranium using novel temperature sensitive urea-formaldehyde resin: adsorption and fast regeneration. *Sci. Total Environ.* **2020,** 735, Art. N° 139399.
36. Liang, L.; Lin, X.; Liu, Y.; Sun, S.; Chu, H.; Chen, Y.; Liu, D.; Luo, X.; Zhang, J.; Shang, R., Carboxymethyl konjac glucomannan mechanically reinforcing gellan gum microspheres for uranium removal. *Int. J. Biol. Macromol.* **2020,** 145, 535-546.
37. He, D. X.; Tan, N.; Luo, X. M.; Yang, X. C.; Ji, K.; Han, J. W.; Chen, C.; Liu, Y. Q., Preparation, uranium (VI) absorption and reuseability of marine fungus mycelium modified by the bis-amidoxime-based groups. *Radiochim. Acta* **2020,** 108, (1), 37-49.

38. Amesh, P.; Venkatesan, K. A.; Suneesh, A. S.; Samanta, N., Diethylenetriamine tethered mesoporous silica for the sequestration of uranium from aqueous solution and seawater. *J. Environ. Chem. Eng.* **2020,** 8, (4), Art. N° 103995.
39. Kolhe, N.; Zinjarde, S.; Acharya, C., Removal of uranium by immobilized biomass of a tropical marine yeast *Yarrowia lipolytica*. *J. Environ. Radioact.* **2020,** 223-224, 106419-106419.
40. Persson, I., Hydrated metal ions in aqueous solution: How regular are their structures? *Pure Appl. Chem.* **2010,** 82, (10), 1901-1917.
41. Li, K.; Li, M.; Xue, D., Solution-phase electronegativity scale: Insight into the chemical behaviors of metal ions in solution. *J. Phys. Chem. A* **2012,** 116, (16), 4192-4198.
42. Xu, H. F.; Xu, D. C.; Wang, Y. F., Natural indices for the chemical hardness/softness of metal cations and ligands. *ACS Omega* **2017,** 2, (10), 7185-7193.
43. Marcus, Y., *Ion Properties*. Marcel Dekker, Inc.: New York, NY, 1997; p 259.
44. Wagman, D. D.; Evans, W. H.; Parker, V. B.; Schumm, R. H.; Nuttall, R. L. *Selected Value of Chemical Thermodynamic Properties - Compounds of Uranium, Protactinium, Thorium, Actinium, and the Alkali Metals*; U.S. Department of Commerce, National Bureau of Standards: Gaithersburg, M.D., USA, 1981; p 149 pp.
45. Gustafsson, J. P. *Visual MINTEQ*, ver. 3.1; KTH, Royal Institute of Technology: Stockholm, Sweden, 2013.
